# A model-based cost-effectiveness analysis of prescribing by dietitians and therapeutic radiographers in England

**DOI:** 10.1007/s10198-025-01813-3

**Published:** 2025-07-03

**Authors:** Saeideh Babashahi, Nicola Carey, Karen Stenner, Kath Hart, Yogini Jani, Judith Edwards, Natalia Hounsome

**Affiliations:** 1 Department of Global Health and Infection, Brighton and Sussex Medical School, University of Brighton and University of Sussex, Brighton, BN1 9PX UK; 2 Centre for Rural Health Sciences, University of the Highlands and Islands, Inverness, UK; 3 Institute for Health Research, University of Bedfordshire, Luton, LU2 8LE UK; 4 School of Biosciences & Medicine, Faculty of Health and Medical Sciences, University of Surrey, Guildford, UK; 5 Centre for Medicines Optimisation Research and Education, UCLH NHS Foundation Trust and UCL School of Pharmacy, London, UK; 6 Centre for Primary Care, Wolfson Institute of Population Health, Barts and The London School of Medicine and Dentistry, Queen Mary University of London, London, UK

**Keywords:** Economic evaluation modelling, Cost-effectiveness analysis, Decision tree, Non-medical prescribing, Dietitian, Therapeutic radiographer, 100, 115, H51

## Abstract

Non-medical prescribing (NMP) was introduced into the UK healthcare system and other countries to improve patient care and facilitate better access to medicine. However, very few studies have evaluated the cost-effectiveness of the prescribing authorities granted to certain healthcare professional groups. This study aims to evaluate the cost-effectiveness of prescribing by dietitians and therapeutic radiographers in England. A model-based cost-effectiveness analysis was conducted to evaluate the services provided by dietitian and therapeutic radiographer prescribers compared to services delivered by dietitian and therapeutic radiographer non-prescribers in terms of direct and indirect costs and effectiveness outcomes, e.g. quality-adjusted life year (QALY) and patient satisfaction, from the National Health Service (NHS) perspective. Unit costs were obtained from the NHS National Reference Costs 2021-22. Sensitivity analyses were performed to assess the robustness of the model parameters. The mean costs associated with NMP were higher for prescribers than non-prescribers due to training costs and consultation time to manage prescriptions. However, these costs were compensated by higher referrals by non-prescribers to other specialists for prescribing. NMP in either profession was perceived as positive by patients. Differences in QALY were not statistically significant among patients managed by prescribers and non-prescribers for either profession. Results were sensitive to the model assumptions and parameters. Our estimates suggest NMP might save £64,269 over five years per dietitian prescriber and £16,570 per therapeutic radiographer prescriber. Despite uncertainties around the cost-effectiveness of NMP, it may save money with minimal or no changes in quality-of-life outcomes for patients being managed by either profession.

**Supplementary Information:**

The online version contains supplementary material available at 10.1007/s10198-025-01813-3.

## Introduction

Globally, the ageing population has led to an increased demand for and access to healthcare services, including medicines [1, 2]. As such, national statistics in England show that the total number of medications dispensed increased from 1.02 billion in 2016/17 to 1.04 billion in 2021/22 [3, 4]. Similarly, in the United States, utilisation of medications grew 4.8% between 2021 and 2022 [5]. The age-related disease burden and the existence of multiple morbidities might result in complex treatments [6] and a higher prevalence of polypharmacy for many patients. In response to these ongoing challenges and the global healthcare workforce crisis [7], new sustainable approaches and models of service provision are required to provide better and more timely access to medicine [8, 9].

Evidence demonstrates that allied healthcare professionals (AHPs), the third largest workforce in the National Health System (NHS), have a fundamental role in supporting transformational and sustainable changes in the UK healthcare system [10–12]. AHPs are encouraged to work at an advanced level in a range of healthcare settings to deal with some of the aforementioned healthcare challenges [8]. Prescribing by AHPs such as dietitians and therapeutic radiographers (TRs), commonly known as non-medical prescribing (NMP), can support the development of such advanced roles for AHPs and create opportunities for innovative and timely services to maintain patients’ better access to medicine while supporting safe and effective prescribing practices [9, 12–20].

Prescribing rights were initially granted to nurses and pharmacists and then extended to a number of AHPs [21, 22] in the UK and some other countries, e.g., the United States, Canada, Australia, New Zealand [12, 14, 23]. NMP was first introduced in the UK for district nurses and health visitors in 1999 [24]. It then came into effect for all registered nurses in 2001 and for pharmacists in 2003 [18, 21, 25]. Since then, these prescribing roles have been gradually extended to other healthcare professionals, including AHPs in primary and secondary care settings in the UK [26, 27]. For a review of the history and evolution of prescribing rights given to healthcare professionals in the UK and other countries, see other studies [18, 23, 28].

Originating in the UK, the term ‘NMP’ refers to the prescriptive authorities awarded to nurses, pharmacists and AHPs (e.g. TRs, dietitians) who have completed an accredited programme of education delivered by a higher education institution [29, 30]. The programme, mainly funded by the NHS or the employing organisation, is delivered via a variety of methods (often hybrid), including classroom teaching, one-to-one instruction, e-learning, self-directed learning and personal study time [31]. A NMP training programme typically takes six months to complete, and it consists of a minimum of 26 days of taught learning and 12 days of supervised prescribing practice within the clinical environment for the trainees [30, 32–34].

In the UK, independent prescribing and supplementary prescribing are different approaches that can be used by non-medical prescribers [25]. Independent prescribers are responsible for clinical decision-making, including prescribing for each patient within their scope of practice and relevant regulations [35, 36]. Supplementary prescribers may prescribe using a patient-specific clinical management plan, agreed upon by the independent prescriber, doctor or dentist and the patient [37–39].

Since 2016, dietitians have been authorised to train as supplementary prescribers and TRs as independent prescribers [39–41]. Although evidence suggests that prescribing by AHPs is perceived as safe with no adverse patient care outcomes [12–14], there is still little evidence regarding the value for money of prescribing authorities granted to many AHP groups, such as dietitians and TRs [23]. Given the importance of NMP practice by healthcare professionals, particularly AHPs working in different healthcare settings, it will be beneficial to evaluate the costs, effectiveness and cost-effectiveness of prescribing rights given to these professionals [12, 23, 42]. This economic evaluation study was conducted as part of a larger research project, ‘the evaluation of supplementary prescribing by dietitians and independent prescribing by radiographers’ (TRaDiP project), funded by the National Institute for Health and Care Research (NIHR) Policy Research Programme [43]. This aspect of the study aims to assess the cost-effectiveness of dietitian supplementary prescribing and TRs independent prescribing in England using a decision tree model.

## Methods

### Study sample and setting

This economic evaluation was undertaken as part of the TRaDiP project (see Supplementary Materials, Appendix 1, for a brief overview of TRaDiP project design), which involved the recruitment of dietitian supplementary prescribers (D-SPs), TR independent prescribers (TR-IPs), dietitian non-prescribers (D-NPs), TR non-prescribers (TR-NPs) and patients managed by these professional groups across eight sites in seven geographical locations in England [43].

### Model structure

**Fig. 1:**
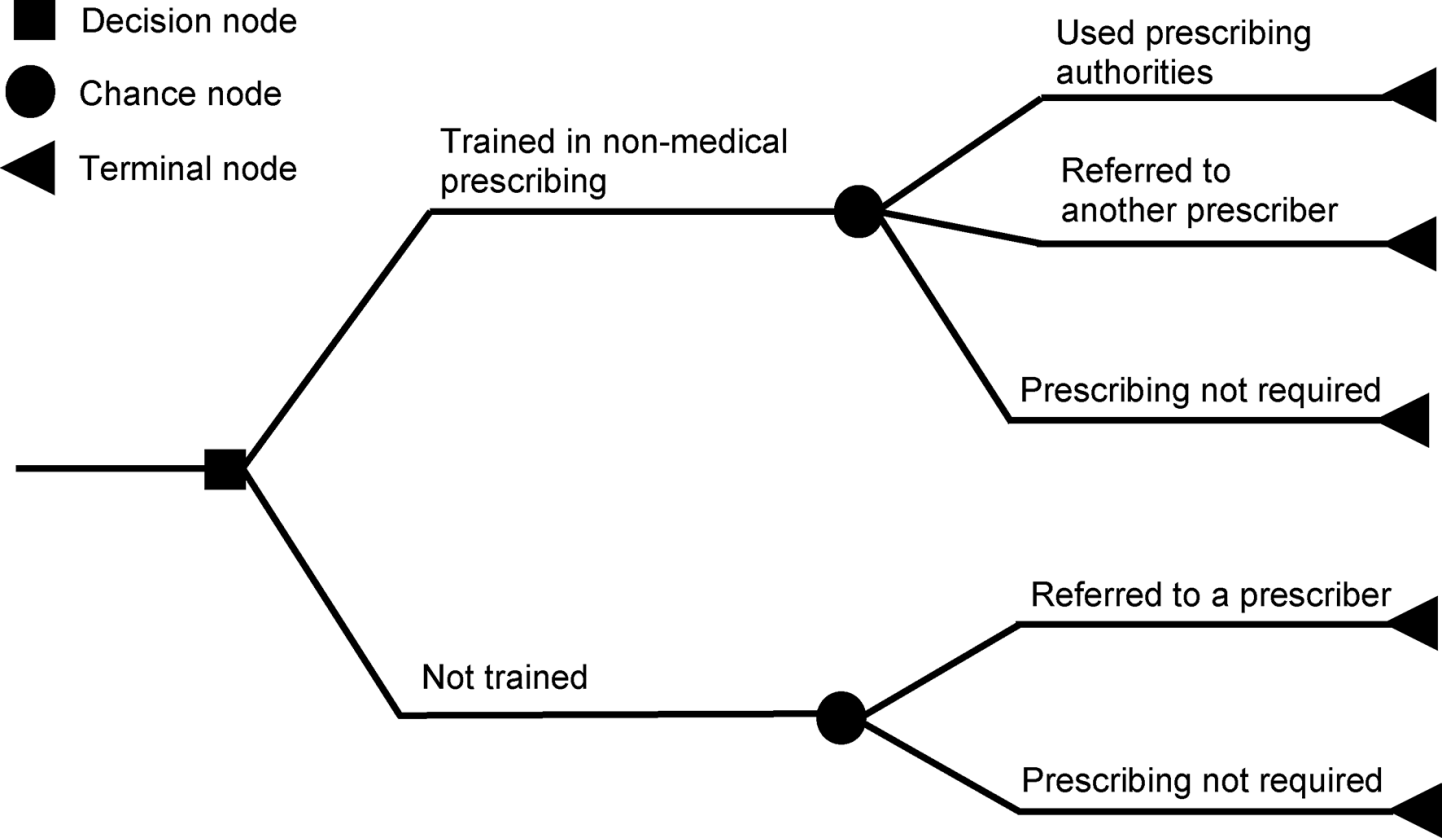
Decision tree illustrating the model. Note: The services provided by non-medical healthcare professionals who are trained in non-medical prescribing (who are then authorised to prescribe within their scope of practice) are compared with services delivered by non-prescribers in terms of costs and effectiveness outcomes (e.g. patient satisfaction, quality-adjusted life years). The same model applied to both dietitians and therapeutic radiographers. For more information, see the model structure in the methods section

A decision tree model was developed in MS Excel 2019 (see Fig. 1) to evaluate costs and patient-reported outcomes of services provided by D-SPs and TR-IPs compared to those delivered by D-NPs and TR-NPs. The model considered two decision options: to train or not to train healthcare professionals in NMP. The trained professionals (D-SPs and TR-IPs in this study) may or may not use their prescriptive authorities for a number of reasons and instead refer patients to other prescribers (e.g. General Practitioners, GPs) for prescribing purposes. The role of trained professionals in NMP extends beyond managing medicines, and not all consultations require managing and reviewing prescriptions. There may also be a delay in prescribing while the qualification is formally annotated by the regulatory body, and some qualified prescribers stop prescribing due to a change in role or other reasons. Therefore, we incorporated these relevant assumptions and probabilities into the model. Professionals not trained in NMP (in this study, D-NPs and TR-NPs) refer patients to a prescriber when prescription management is required. We assumed safety is consistent between both arms of the model.^1^ The model structure was the same for both professions, although some inputs used to inform the analysis were profession-specific. The model was designed to analyse the cost-effectiveness of prescribing rights by either profession from the England National Health Service perspective. The 2022 Consolidated Health Economic Evaluating Reporting Standards (CHEERS) were followed [44].

### Model parameters, data collection tools and data sources

Model parameters (e.g. costs, effectiveness outcomes, probabilities) were obtained from various sources, including the primary data collected within the study and a scoping review published elsewhere (for model parameters, see Appendix 2) [23, 43]. The TRaDiP project involved prescriber surveys, self-report audits and economic assessment questionnaires completed by D-SPs, D-NPs, TR-IPs and TR-NPs to explore prescribing activities, NMP uptakes and trends, as well as surveys of patients managed by prescriber and non-prescriber professionals across eight sites in England. Prescriber surveys with NMPs included data about costs associated with undertaking the prescribing programme, including course fees, funding arrangement, mode of study, number of days of supervised learning in practice, hours spent with practice supervisor, additional payments from employer and out-of-pocket expenses (e.g. travel, fuel, accommodation, study books and materials). The economic assessment questionnaires included data on patients’ contact per week, percentage of patients required to manage their prescription, percentage of time spent on prescribing-related activities, percentage of patients referred for prescription to other prescribers, training course duration, other relevant out-of-pocket expenses. Patients’ referrals for prescription purposes were drawn from self-report audits for each profession. The patients’ questionnaires included data on how respondents usually received their prescription for the consulted condition, prescriber information, waiting time to obtain a prescription, patients’ satisfaction, and their experience of the consultation with the D-SPs and TR-IPs, as well as the overall health status of patients. Patients also completed the validated EQ-5D-5 L quality of life questionnaire developed by the EuroQoL Research Foundation [45]. Also, the Health and Care Professional Council^2^ and course websites were used to gather information about prescribing programmes (e.g. course fee, course duration) for dietitians and TRs in England in 2021, delivered by different higher education institutes (Appendix 3.1 and Appendix 3.2) [30].

A within-study analysis was conducted to populate the model with costs, probabilities, effectiveness outcomes and estimates of uncertainty. Unit costs were taken from the NHS National Reference Costs and the Personal Social Services Research Unit Costs for the Health and Social Care report [46, 47]. Staff salaries were estimated using the NHS 2021/22 pay scales [48]. More information about costs and effectiveness outcomes is provided in the following sub-sections.

### Costs Estimation

Costs were measured in 2021 pounds sterling. Direct costs included training-related expenses such as training course fees, employer-paid additional study time, out-of-pocket expenses paid by trained professionals, and costs of personal study time. We considered different cost scenarios (see Appendix 4 for study assumptions). Indirect costs were incorporated in the analysis by estimating the opportunity costs of the time off work to complete the training course (i.e. the monetary value of the work time lost). Other relevant indirect costs included costs of referrals to other prescribers (e.g. GPs) and the cost of consultations (and prescribing-related activities) for both prescriber and non-prescriber groups. Each of the cost categories is described in the following sub-sections.

The cost analyses were conducted using one-year and five-year post-training time horizons. The duration of the base-case cost-effectiveness analysis was aligned with the duration of the study (one year) for which data were collected. This was based on the duration of the prescribing training programmes, with the majority completed within one year. We extrapolated the costs over the five-year time horizon to reflect that the training costs were incurred upfront and assess that prescribing by both professions can be cost-saving in the short and medium terms. To estimate costs for the five-year time horizon scenario, the costs associated with the training programme were only considered in the first year, with the cost of referral, consultation (and prescribing-related activities) included for all five years. In line with the recommendations of the National Institute for Health and Care Excellence (NICE), the costs associated with referral, consultation, and prescribing-related activities that occurred in years two to five were adjusted at a rate of 3.5% [49, 50].

### Cost of training in non-medical prescribing

Training costs included course fees, employer-paid additional study time and out-of-pocket expenses associated with attending the course (e.g. travel, accommodation, study materials and personal study time). Out-of-pocket expenses were included in the cost since these could potentially be reimbursed by the employer. The data on employer-paid additional study time, staff pay grades, and out-of-pocket expenses for the study sample were collected within the study. Additional personal study time was costed using the NHS pay scales 2021–2022 [48]. Not all patients require a prescription or manage their medication. Therefore, two scenarios were considered when calculating the training costs: (1) the average cost per patient seen and (2) the average cost per patient required to manage their medication.

### Cost of consultation and prescribing-related activities

Spending time on prescribing activities (e.g. reviewing medication) means that non-medical prescribers might need to spend more time managing medications and cannot use this time for other patients (compared to the non-prescribers). Therefore, costs associated with additional time spent reviewing the medication were accounted for by the prescriber group in the cost-effectiveness analysis. To calculate the costs associated with consultation and prescribing-related activities, we used primary data (e.g. the number of patients seen or required to manage their medication, consultation time with patients, preparation time for prescribing, and time spent writing the prescription) collected within the TRaDiP project. Consultation costs were estimated using the NHS pay scales 2021–2022 [48].

### Cost of referrals

Both prescribers and non-prescribers in the two professions may refer patients to other prescribers for prescribing purposes. A referral for a prescription may require a consultation with another healthcare professional (e.g., GP, hospital consultant) face-to-face, via telephone, or online. This means that this consultation cannot be used for other patients and should be included in the costs. The data on the proportion of patient referrals was obtained from the study sample. We elicited the relevant potential groups of professions to which the patients were referred for prescribing purposes by either profession according to audit data and specialities of the case sites in the study.

A subsequent list of services and their average national unit costs were gathered for costing from the NHS National Reference Cost Dataset 2021-22 [47], representing different specialities, e.g. GPs, consultants or other relevant healthcare professionals led by consultants providing face-to-face and non-face-to-face consultations to patients referred by both professions. We used medical and clinical oncology services for a range of cancers covered by the case sites in the study for the two professions (Appendix 5).

### Effectiveness outcomes

The primary effectiveness outcome in the study was patient health-related quality of life (measured in terms of quality-adjusted life year, QALY^3^), with patient waiting time to obtain a prescription, patient overall experience of the consultation and patient overall satisfaction representing the secondary effectiveness outcomes of interest. Due to the heterogeneity of patients (with a range of health conditions managed by two different professions) in the study, we were not able to consider disease-specific health and clinical outcomes in the analysis. Also, patients’ overall experience and satisfaction with the consultation they received as well as their waiting time to obtain a prescription, were identified as other important effectiveness outcomes in this context by the relevant literature [12, 51].

The data regarding the time to obtain a prescription from non-medical prescribers (in this study, D-SPs and TR-IPs) was collected from the study sample with the following categories: ‘less than 5 minutes’, ‘5 to 10 minutes’, ‘10 to 20 minutes’, ‘20 to 30 minutes’, ‘more than 30 minutes’, ‘the next day’, ‘several days or more’, and ‘don’t know’. In the case of referrals to other specialists, the time categories were: ‘on the same day’, ‘1–3 days’, ‘4–6 days’, ‘1–2 weeks’, ‘up to a month’, ‘more than a month’.

Similarly, patient overall satisfaction with their consultation and patient overall experience of the consultations were collected based on the study sample with the following categories: ‘strongly disagree’, ‘disagree’, ‘no opinion’, ‘agree’, and ‘strongly agree’. Patient health-related quality of life was measured using the EuroQoL questionnaires [52, 53]. The EQ-5D-5 L, a commonly used generic patient-reported outcome measure, was used in the study. It has five dimensions (mobility, self-care, usual activities, pain and discomfort, anxiety and depression) with five response levels (no problem, slight problem, moderate problem, severe problem and extreme problem) [52, 53]. QALYs were then calculated using the value sets for England [54]. QALY values vary from 1 to 0, with 1 indicating a perfect health state and 0 indicating death.^4^

### Cost-effectiveness analysis

We conducted a cost-effectiveness analysis to evaluate incremental changes in costs and effectiveness outcomes for services provided by prescribers versus non-prescribers in both professions (i.e. dietitians and TRs). The incremental cost-effectiveness ratio (ICER) was calculated as the difference in costs between services provided by prescribers and non-prescribers (incremental cost, ΔC) divided by the difference in effectiveness outcomes (incremental effect, ΔE).

Costs included in the analysis consisted of the cost of training, the cost of consultation (and prescribing-related activities for prescribers) and the cost of referrals to other prescribers. Effectiveness outcomes included QALY, patient waiting times to obtain a prescription, patient satisfaction, and patient experience of consultation. In the analysis of waiting time, the denominator was multiplied by -1 to reflect the fact that a lower waiting time represents a better outcome.

In the cost-effectiveness analysis using QALY (also referred to as cost-utility analysis), the probability of NMP being cost-effective was estimated using the net monetary benefit (NMB) approach. NMB represents the monetary value of extra gains in QALY associated with the intervention (in this study, training professionals in NMP) for a given willingness to pay (WTP).

$$\mathrm{NMB}=\mathrm{WTP}\times\Delta\mathrm{E}-\Delta\mathrm{C}$$

NMP is considered to be cost-effective if the following decision rule applies:

$$\mathrm{WTP}\times\Delta\mathrm{E} -\Delta\mathrm{C}>\mathrm{0}$$

We explored the probability that NMP is cost-effective at £30,000 per QALY considered by NICE for the UK (including England) context [49].

### Statistical analysis

The effectiveness outcomes (e.g. QALY, patient satisfaction) were adjusted for covariates using a mixed-effects linear regression model with ‘study case site’ as a random effect to account for the heterogeneity of the sample. Covariates included in the model as fixed effects were age, gender and general health status. A nonparametric bootstrap was used with 5,000 replications to obtain mean differences in effectiveness outcomes and confidence intervals. This analysis was conducted using STATA/IC 16 software.

We performed univariate sensitivity analyses (in the form of tornado charts) to test the robustness and identify the most influential variables influencing the cost-effectiveness of NMP practice by either profession. Monte Carlo simulation was performed with 5,000 iterations through the model sampling across all distributions simultaneously to conduct probabilistic sensitivity analysis and assess the robustness of the cost-effectiveness analysis in MS Excel 2019. The analysis included cost-effectiveness scatter plots showing the mean differences in costs and effectiveness outcomes across the prescriber and non-prescriber groups. In addition, the results were presented in terms of cost-effectiveness acceptability curves that indicated the probability of training the two professions in NMP being cost-effective over a range of WTP values.

### Ethical considerations

Ethical approvals were obtained from the University of Surrey Ethics Committee (UEC 2019-076) and the London-Camberwell St Giles Research Ethics Committee (21/LO/0316). Participation was voluntary, and participants were free to withdraw at any time. Where possible, study information was sent a couple of weeks in advance to all potential patient participants. Each site also advertised the study prior to and during data collection. All participants gave consent prior to the data collection.

## Results

### Summary of general characteristics of the study participants

A total of 9 case sites were recruited comprising matched D-SPs/D-NPs and TR-IPs/TR-NPs (three dietetic and six therapeutic radiography sites), one (case study 4) of which withdrew from the study. Participants were based in different geographical regions in England, with the largest representation from the Midlands (21.7%, *n* = 20) and the lowest from the Southeast (6.5%, *n* = 6). Table 1 provides detailed information about the eight sites in the study.

**Table 1:** General characteristics of case study sites

Case site	Profession	Status	Job Title	Setting	Location in England*
2	Dietitian	D-SP	Lead Intestinal Rehabilitation Dietitian	Specialist acute NHS hospital (inpatient/outpatient)	London
		D-NP	Advanced Specialist Dietitian		
5		D-SP	Lead Clinical Dietitian	NHS community trust (outpatient)	West Midlands
		D-NP	Community Diabetes Dietitian		
7		D-SP	Specialist Renal Dietitian	Major acute specialist hospital (inpatient/outpatient)	Northeast & North Cumbria
		D-NP	Specialist Renal Dietitian		
1	TR	TR-IP	Review Therapeutic Radiographer	Major acute NHS hospital (outpatient)	West of England
		TR-NP	Review/Treatment Radiographer		
3		Trainee TR-IP	Review Therapeutic Radiographer	Major acute NHS hospital (outpatient)	West of England
		TR-IP	Review Therapeutic Radiographer		
6		TR-IP	Advanced Practitioner Therapeutic Radiographer	NHS tertiary cancer centre (outpatient)	Oxford & Thames Valley
		TR-NP	Review/Treatment Therapeutic Radiographer		
8		TR-IP	Macmillan Specialist Radiographer	Acute NHS hospital (outpatient)	Southwest
		TR-NP	Review/Treatment Therapeutic Radiographer		
9		TR-IP	Advanced Review Therapeutic Radiographer	Major acute NHS hospital (outpatient)	Northwest Coast
		TR-NP	Review Therapeutic Radiographer		

Note: TR: Therapeutic radiographer; D-SP: dietitian supplementary prescriber; D-NP: dietitian non-prescriber; TR-IP: therapeutic radiographer independent prescriber; TR-NP: therapeutic radiographer non-prescriber; *According to Health Innovation Network regions (https://www.england.nhs.uk/ourwork/part-rel/healthinnovationnetwork/)

In sites 1, 2, 5 to 9, an independent/supplementary prescriber was matched with a non-prescriber. At case site 3, a single TR completed data collection as a trainee TR-IP (i.e., before qualifying as a prescriber) and after qualifying as a TR-IP. Matching was primarily based on the type of service, clinical role and care setting. Other considerations for matching included patient demographics and Agenda for Change banding [49]. Data used in the study analysis were gained from 92 prescriber professionals who completed the prescriber surveys (see the Methods section), of whom 54 (58.7%) were TRs and 38 (41.3%) were dietitians. A total of 513 self-report audits were completed by 169 dietitians and 344 TRs. A total of 180 patients completed the patient questionnaires (49 patients managed by dietitians and 131 by TRs). The economic assessment questionnaires were completed by eight D-SPs/TR-IPs and eight D-NPs/TR-NPs.

### Cost of training in non-medical prescribing

A summary of the costs and assumptions used to estimate the cost of training for both professions is provided in Table 2. The average fee for the accredited prescribing programmes in England was £1,801 (range £1,200–£3,500) for dietitians and £1,951 (range £1,070–£4,000) for TRs. The employers paid, on average, six days (range 1–11 days) of additional study time for dietitians, which was estimated at £797 per trainee (range £133–£1,400). For TRs, the employers paid, on average, seven days (range 2–14 days) of additional study time, which was estimated at £951 per trainee (range £266–£1,859).

**Table 2:** Non-medical prescribing training courses and associated costs

	Dietitians		Therapeutic radiographers	
	Mean	Range	Mean	Range
**Training programme**				
Course fee (£)	1,801	1,200–3,500	1,951	1,070–4,000
Course duration (months)	7	3–13	8	3–13
Employer-paid additional study time (days)	6	1–11	7	2–14
Pay value, salary range (£)	48,456	44,606–52,305	48,456	44,606–52,305
Cost of employer-paid additional study time (£)	797	133–1,400	951	266–1,859
Taught days in the training programme (days) *	26	NA	26	NA
Days of supervised learning completed for the programme (days) *	12	12–13	12	12–13
Cost of the time off work to complete the course – staff backfill (excluding personal study times) (£) **	2,522	NA	2,522	NA
**Out-of-pocket expenses** (OOPs, paid by trainees)				
Travel expenses (£)	132	10–400	209	36–600
Textbooks and study materials (£)	105	10–400	62	20–150
Other OOP expenses (£)	193	30–400	45	25–60
Personal study time (days)	29	7–60	27	4–60
Personal study time (£)	3,791	929–7,965	3,584	531–7,965

Note: * As a requirement, each trainee had to complete 26 taught and 12 supervised days. The costs of supervised days are already included in the non-medical prescribing training programme fee. The number of taught and supervised days was the same for both professions. ** The cost associated with time off work to complete the non-medical prescribing course was estimated using the number of ‘taught’ and ‘supervised’ days. NA: Not Applicable; OOP: Out-of-pocket expenses

In both professions, each trainee spent 26 taught days of training and 12 days of supervised learning as a minimum requirement. Based on evidence gained from the two professions, we deduced the trainees spent approximately 50% of their work time on the training course. Therefore, the time off work to complete the course was costed using the average pay band for each profession in the study sample (average of £48,456, range £44,606–£52,305) multiplied by half the time spent on the programme. This was £2,522 per trainee based on the ‘required’ time to complete the course for each profession (Table 3).

**Table 3:** Training costs per prescriber and per patient contact

	Dietitians		Therapeutic radiographers	
	Mean	Range	Mean	Range
**Scenario 1: Including OOP expenses**				
Average training cost per prescriber (£)*	9,341	4,834–16,654	9,324	4,470–17,223
Average training cost per patient contact (£)	21	20–23	10	10–16
Average training cost per patient contact required to manage prescriptions (£)	34	32–37	16	15–26
**Scenario 2: Excluding OOP expenses**				
Average training cost per prescriber (£)*	5,120	3,855–7,489	5,425	3,858–8,447
Average training cost per patient contact (£)	12	10–16	6	5–14
Average training cost per patient contact required to manage prescriptions (£)	19	17–26	9	7–22

Note: * for detailed costs, please see Table 2. For detailed information about patient contacts, see Appendix 5 in Supplementary Materials

Based on the study sample, the out-of-pocket (OOP) expenses paid during training by dietitians were, on average, £132 (range £10–£400) for travel, £105 (range £10–£400) for textbook and study material, and £193 (range £30–£400) for other OOP expenses. (Table 3). Therapeutic radiographers paid, on average, £209 for travel (range £36–£600), £62 for textbook and study material (range £20–£150), and £45 for other OOP expenses (range £25–£60). In terms of personal study time, an average of 29 days (range 7–60 days) and 27 days (range 4–60) was spent by dietitians and TRs, respectively.^5^ The personal study time was estimated to cost on average £3,791 for each dietitian (range £929–£7,965) and £3,584 (range £531–£7,965) for each TR (Table 3).

### Training-related costs per patient

The cost of training per prescriber and per patient contact was estimated using two costing scenarios, as shown in Table 4. In the base-case scenario, the OOP expenses were included in the analysis as it was assumed that trainees may receive reimbursement for their OOP expenses. In this scenario, the mean cost of training was £9,341 per dietitian (range £4,834–£16,654) and £9,324 per TR (range £4,470–£17,223). In the second scenario, the OOP expenses were assumed to be paid by trainees and, therefore, were excluded from the analysis. The mean training cost, excluding OOP expenses, was £5,120 per dietitian prescriber (range £3,855–£7,489) and £5,425 per TR (range £3,858–£8,447) (the number of patient contacts managed by dietitians and TRs is summarised in Appendix 6).

**Table 4:** Time spent on prescribing-related activities

Activity	Dietitians		Therapeutic radiographers	
	Mean	Range	Mean	Range
Communicating with patients	23%	20–25%	43%	25–70%
Reviewing medication	28%	25–30%	33%	5–85%
Consulting with colleagues	55%	10–100%	13%	10–20%
Writing notes	10%	NA	28%	15–45%

Note: NA: Not Applicable (due to a lack of data on the range). The percentages are reported out of the overall time (100%) a prescriber in either profession spends on prescribing-related activities, including communicating with patients, reviewing medication, consulting with colleagues and writing notes

The average training cost per patient contact was £21 (range £20-£23) with OOP expenses and £12 (range £10-£16) without OOP expenses for each dietitian prescriber. Whereas the average training cost per patient contact was £10 (range £10–£16) with OOP expenses and £6 (range £5–£14) without OOP expenses for each TR prescriber. Since not all patients required medicine management, an additional analysis was conducted to estimate the costs per patient contact requiring a prescription. The mean training cost per contact required to manage a prescription (i.e. issuing new prescriptions, reviewing existing prescriptions and de-prescribing) was £19 (range £17–£26) excluding OOP expenses and £34 (range £32–£37) including OOP expenses for dietitian prescribers. The mean training cost per consultation required to manage a prescription was £9 (range £7–£22), excluding OOP expenses and £16 (range £15–£26) including OOP expenses for therapeutic radiographer prescribers (Table 3).

### Costs of consultations and prescribing-related activities

Table 4 provides data for time spent on prescribing-related activities (i.e. communicating with patients, writing notes, reviewing medication and consulting with colleagues). Prescriber groups were more likely to consider changes in prescribing^6^ than non-prescribers. On average, D-SPs spent 28% and TR-IPs 33% of their work time reviewing medications. This estimate was used to cost the additional consultation times required by prescriber groups to manage the medications. The average cost of consultations which required prescribing was £157 (range £125–£190) for D-SPs and £116 (range £69–£168) for TR-IPs. For consultations which did not require prescribing, the estimated cost of consultation was £123 (range £98–£149) for D-NPs and £87 (range £52–£127) for TR-NPs. For a list of face-to-face and non-face-to-face consultations for both professions and the unit costs obtained from the NHS reference cost 2021-22 [48], see Appendix 5.

### Costs of referrals to other prescribers

The number of patient contacts managed by dietitians and therapeutic radiographers, as well as referrals to other specialists for prescribing purposes, are summarised in Appendix 6. D-SPs used their prescribing qualification in 64% of the consultations; this was 87% for TR-IPs.

On average, both D-SPs and D-NPs had nine patient consultations per week (range 5–15). The average number of consultations required to manage prescriptions by D-SPs was six (range 3–9). The percentage of patients referred to other prescribers was 2% in the prescriber group and 30% in the non-prescriber group. On average, both therapeutic radiographer independent prescribers (TR-Ips) and therapeutic radiographer non-prescribers (TR-NPs) had 19 patient consultations per week (range 6–38). The average number of consultations required to manage prescriptions by TR-IPs was 12 (range 4–24). The percentage of patients referred to other prescribers was 7% in the prescriber group and 23% in the non-prescriber group. A year of 48 working weeks was assumed to estimate the number of patient consultations annually for the two professions (Appendix 6). The average cost of referral to other healthcare professionals was £188 (£76–£364) for dietitians and £179 (£76–£364) for therapeutic radiographers. For a list of referral services, see Appendix 5.

### Overall costs

The estimated average total cost of NMP (including the cost of training, cost of consultations and cost of referrals to other specialists for prescribing) in the first year following training was £74,820 for D-SPs compared to £79,206 for D-NPs. The average five-year costs were £360,469 for D-SPs and £424,738 for D-NPs. On average, supplementary prescribing by dietitians could save £4,386 per prescriber in the first year following training and £64,269 per prescriber over five years. For therapeutic radiographers, the estimated average total cost of NMP in the first year following training was £121,918 for TR-IPs compared to £117,422 for TR-NPs. The average five-year costs were £613,102 and £629,672 for TR-IPs and TR-NPs, respectively. On average, independent prescribing by therapeutic radiographers would save £16,570 per prescriber over five years.

### Effectiveness outcomes

Table 5 represents the effectiveness outcomes used in this economic evaluation (for non-adjusted values, see Appendix 7).^7^ The EQ-5D-5 L responses for each of the five dimensions (e.g. mobility, self-care, usual activities) for patients managed by prescribers and non-prescribers in both professions are provided in Appendix 8. The mean adjusted QALY was lower in the D-SP group (0.7403, SD = 0.0223) compared to the D-NP group (0.7526, SD = 0.0269) (not significant, p-value = 0.080). For therapeutic radiographers, the mean adjusted QALY was lower in the TR-IP group (0.7299, SD = 0.0250) compared to the TR-NP group (0.7359, SD = 0.0291), although again, this difference was not statistically significant (p-value = 0.207). Although there were small differences in patient experience of consultation and overall experience scores between prescribers and non-prescribers for each profession, these were not significant (all p-values > 0.05) (Table 5).

**Table 5:** Effectiveness estimates used in economic analysis

Assessment	Prescribers		Non-prescribers		Difference in mean	95% CI*
	Mean	SD	Mean	SD		
**Dietitians**						
QALY	0.7403	0.0223	0.7525	0.0269	-0.0122	(-0.0817, 0.0551)
Patient’s overall satisfaction with the consultation	77.32	7.38	76.38	7.63	0.95	(-3.37, 5.26)
Patient’s overall experience of the consultation	65.2	7.39	63.33	5.65	1.87	(-1.93, 5.66)
**Therapeutic radiographers**						
QALY	0.7299	0.0250	0.7359	0.0291	-0.0060	(-0.0816, 0.0686)
Patient’s overall satisfaction with the consultation	79.57	0.96	79.35	0.66	0.22	(-0.0556, 0.5002)
Patient’s overall experience of the consultation	65.69	0.97	66.24	1.09	-0.55	(-0.9140, -0.1867)

Note: Effectiveness outcomes were adjusted for covariates using a mixed-effects model (see Methods). * As the 95% confidence interval (-0.0824–0.0566) contains zero, it indicates that the differences in QALY between the prescriber and non-prescriber groups can be positive as well as negative; however, the differences are not statistically significant

The data on patient waiting time is shown in Appendix 9. Data were primarily derived from 20 patients from the study questionnaire, which included filter questions that narrowed down the sample to *N* = 5 for patients managed by D-SPs and *N* = 3 for those managed by D-NPs. The mean waiting time was 1.67 (SD = 0.6) for D-SPs and 3.7 (SD = 2.3) for D-NPs (Appendix 9). The average patient waiting times for both TR-IPs and TR-NPs were one day with no variation across the sample (*N* = 12 TR-IPs and *N* = 3 TR-NPs). The data on patients waiting time to obtain a prescription was inconclusive due to a very small sample size, and it was not included in the model.

### Cost-effectiveness of non-medical prescribing

The summary of the base-case cost-effectiveness analysis is presented in Table 6. The difference in average total costs was -£10 between D-SPs and D-NPs and £5 between TR-IP-SP and TR-NP. The negative incremental cost between dietetic prescribers and non-prescribers was due to a lower number of referrals to other specialists by D-SPs, which offsets the cost of training. The difference in QALYs was negative (-0.0122 and − 0.0060 for dietitians and therapeutic radiographers, respectively) due to slightly lower patient QALYs in the prescribers group for both professions. The ICER point estimate was £816 per QALY lost for dietitians (supplementary prescribing was less costly but less effective) and -£824 per QALY lost for TRs (independent prescribing was more costly but less effective).

**Table 6:** Results of the cost-effectiveness analyses of non-medical prescribing by dietitians and therapeutic radiographers

Outcome	Total cost, £ mean (SD)	Total effect, mean (SD)	Difference in cost, £ mean (95% CI)	Difference in effect, mean (95% CI)
**Patients managed by dietitians**				
**QALY**				
Prescribers	169 (34)	0.74 (0.02)	-10 (-179, 120)	-0.0122 (-0.0824, 0.0566)
Non-prescribers	179 (69)	0.75 (0.03)		
**Patient’s overall satisfaction with the consultation**				
Prescribers	169 (34)	77.32	-10 (-179, 120)	0.95 (-3.38, 5.26)
Non-prescribers	179 (69)	76.38		
**Patient’s overall experience of the consultation**				
Prescribers	169 (34)	65.20	-10 (-179, 120)	1.87 (-1.93, 6.66)
Non-prescribers	179 (69)	63.33		
**Patients managed by therapeutic radiographers**				
**QALY**				
Prescribers	134 (61)	0.73 (0.03)	5 (-194,183)	-0.0060 (-0.0816, 0.0686)
Non-prescribers	129 (71)	0.74 (0.03)		
**Patient’s overall satisfaction with the consultation**				
Prescribers	134 (61)	79.57	5 (-194,183)	0.22 (-0.0586, 0.5002)
Non-prescribers	129 (71)	79.35		
**Patient’s overall experience of the consultation**				
Prescribers	134 (61)	65.69	5 (-194,183)	-0.55 (-0.9140, -0.1867)
Non-prescribers	129 (71)	66.24		

Note: Costs were rounded to the nearest £

**Fig. 2:**
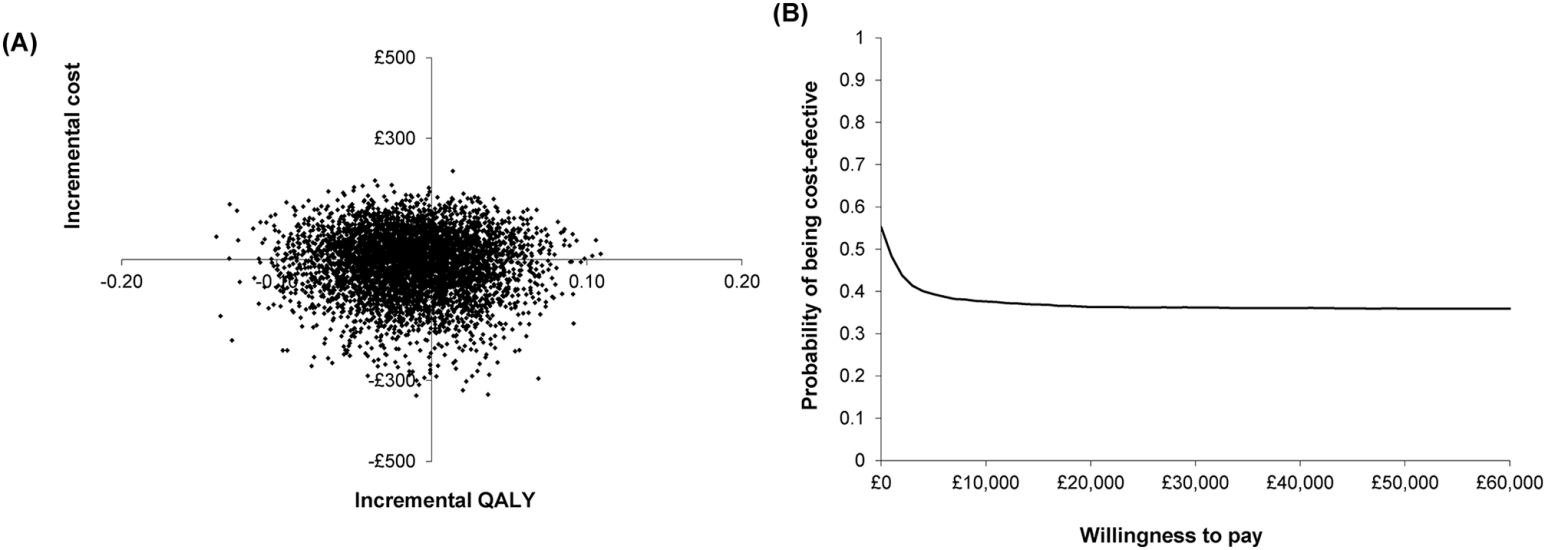
(**A**) Cost-effectiveness plane and (**B**) cost-effectiveness acceptability curve for dietitian prescribers vs. dietitian non-prescribers based on 5,000 Monte Carlo simulations of total costs and QALY (adjusted using the mixed effects model)

Figure 2 shows the cost-effectiveness plane (A) and the cost-effectiveness acceptability curve (B) for services provided by D-SPs compared to those by D-NPs based on 5,000 Monte-Carlo simulations of incremental costs and QALYs. Almost 19% of simulations were in the southeast quadrant (where services provided by D-SPs were less costly and more effective compared to consultations with D-NPs) and 18% in the northeast quadrant (services provided by D-SPs were more costly and more effective). The probability of supplementary prescribing being cost-effective at the NICE threshold of £30,000 was around 37%. This means that there is high uncertainty about the cost-effectiveness of supplementary prescribing by dietitians, although based on our analysis, it may save money in the medium and long terms with very little or no effect on patients’ quality of life.

The cost-effectiveness analysis using both patient satisfaction and patient experience of the consultation as two additional outcome measures resulted in negative incremental cost and positive incremental effectiveness scores (Table 6), indicating that consultations with D-SPs were less costly and more effective than consultations with D-NPs in terms of patient satisfaction. However, there is high uncertainty around this estimate, as shown by Appendix 10 and Appendix 11, where estimates fell in all four quadrants of the cost-effectiveness plane. We do not report ICERs and probabilities of being cost-effective for this outcome since there is no WTP threshold defined for patient satisfaction.

**Fig. 3:**
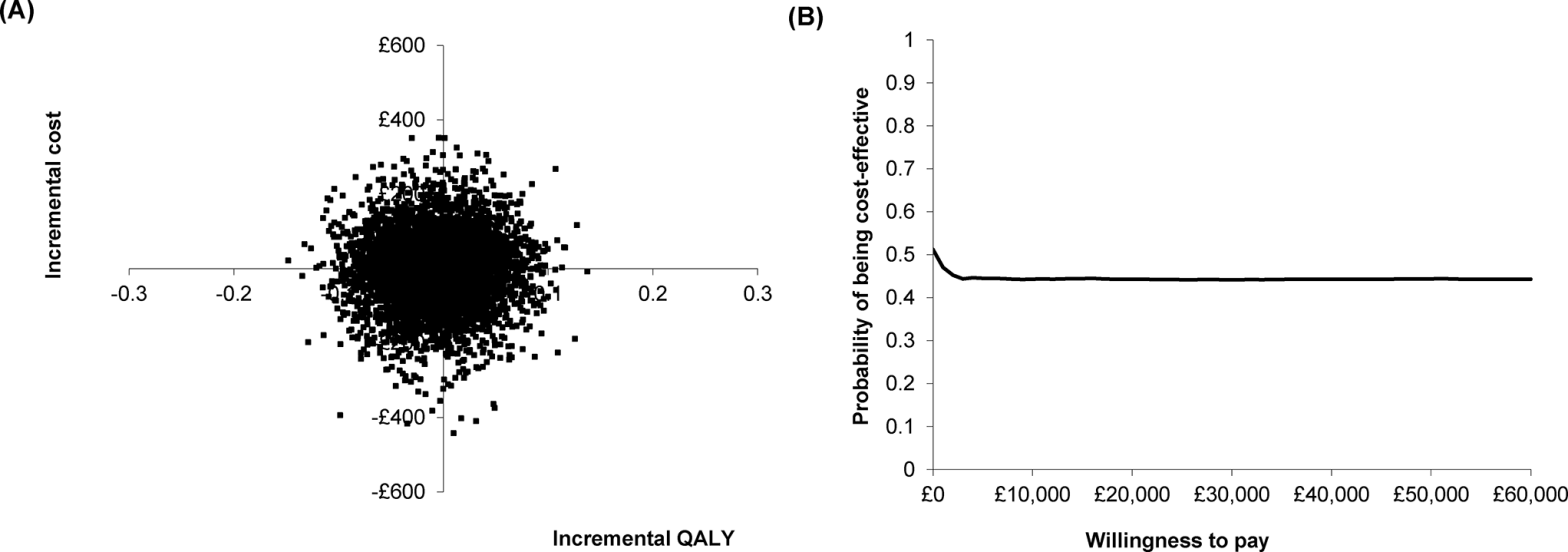
(**A**) Cost-effectiveness plane and (**B**) cost-effectiveness acceptability curve for therapeutic radiographer prescriber vs. therapeutic radiographer non-prescribers based on 5,000 Monte Carlo simulations of total costs and QALY (adjusted using the mixed effects model)

Figure 3 represents (A) the cost-effectiveness plane and (B) the cost-effectiveness acceptability curve for services provided by TR-IPs compared to TR-NPs. As shown in Fig. 3A, 20% of simulations were in the southeast quadrant, where consultations with TR-IPs were less costly and more effective compared to consultations with TR-NPs and 23% in the northeast quadrant (consultations with TR-IPs were more costly and more effective). Figure 3B shows that the probability of independent prescribing being cost-effective at the NICE threshold of £30,000 was around 44%. In summary, there is high uncertainty about the cost-effectiveness of independent prescribing, although it may save money in the long term with little or no effect on patients’ quality of life.

The cost-effectiveness analysis using patient overall satisfaction with consultations resulted in positive incremental cost and positive incremental effectiveness scores (Table 6), indicating that consultations with TR-IPs were slightly more costly and more effective than consultations with TR-NPs in terms of patient satisfaction. However, there was high uncertainty around these estimates, as shown in Appendices 12 and 13. We do not report the ICER point estimate and probability of independent prescribing being cost-effective for this outcome since there is no WTP threshold for patient satisfaction.

**Fig. 4:**
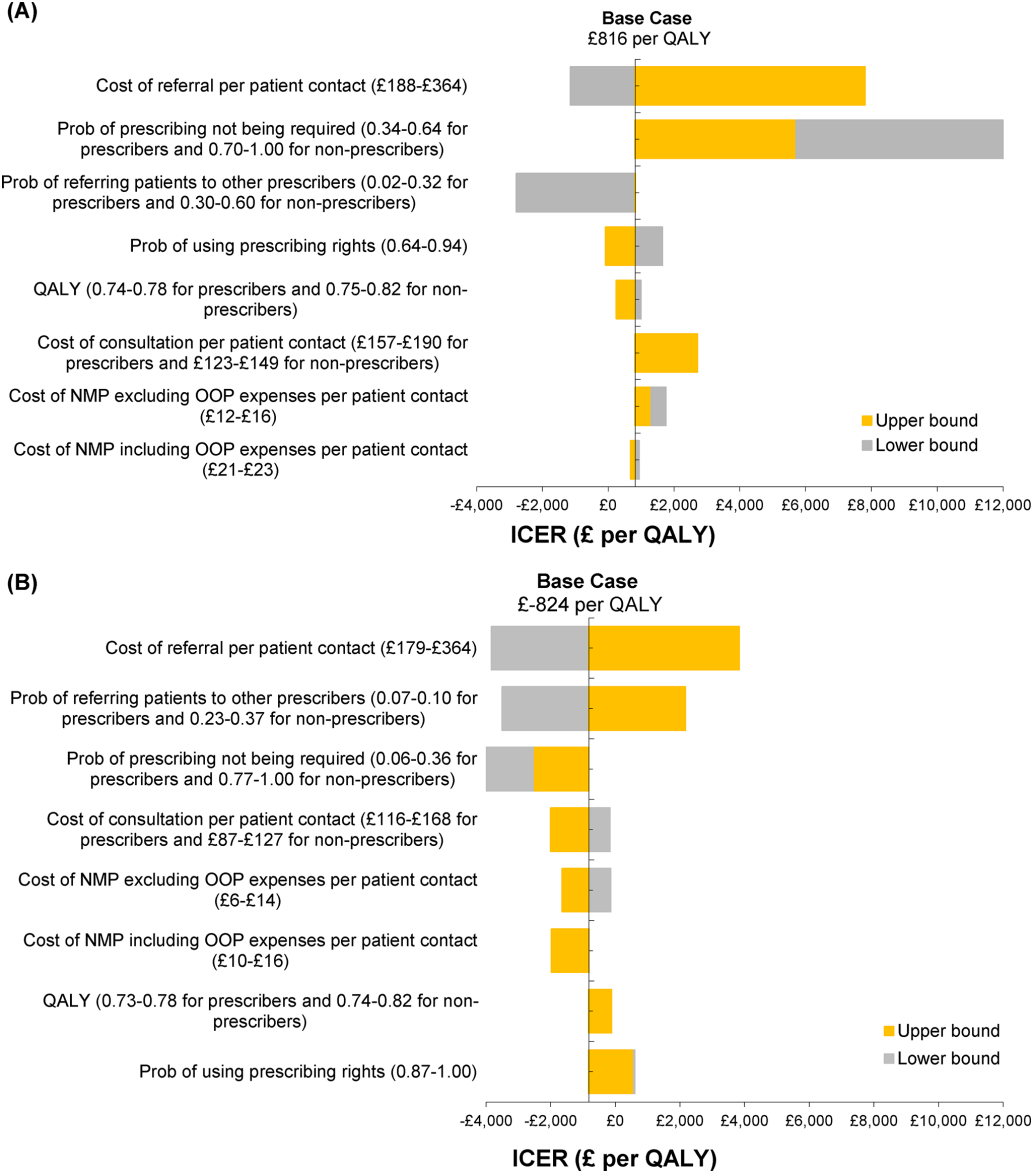
Deterministic sensitivity analysis: (**A**) tornado charts for dietitians (**B**) tornado charts for therapeutic radiographers. Note: Prob: Probability; NMP: Non-medical prescribing; OOP: Out-of-pocket expenses; QALY: Quality-adjusted life year; ICER: Incremental cost-effectiveness ratio. The base case shows the cost per QALY lost. Numbers inside the parentheses show the lower and upper bounds for each parameter for prescribers (and non-prescribers, where relevant). Also, see Appendix 14 and Appendix 15 in Supplementary Materials for more information about the results of deterministic sensitivity analyses in either profession

The tornado sensitivity analysis results for model parameters are represented in Fig. 4 in descending order. Figures 4(A) and (B) show that the most influential parameters for both professions that influence the incremental results are the cost of referral, probability of not being required, probability of referring patients to other prescribers, and probability of using prescribing rights (also see Appendix 14 and Appendix 15 for deterministic sensitivity analyses results).

## Discussion

This is the first model-based economic evaluation study to examine the potential costs and cost-effectiveness of dietitian supplementary prescribing and TR independent prescribing in England. To the best of our knowledge, there are no other studies that directly observed and evaluated the costs, effectiveness and cost-effectiveness of prescribing authorities by dietitians and TRs in the UK or the world. We examined services provided by prescribers compared with non-prescribers in both professions in terms of costs and effectiveness outcomes over a one-year time horizon. Our analysis indicates that NMP by either profession has a low probability of being cost-effective. This is not surprising due to NMP training costs occurring in the first year and the longer consultation times by prescriber groups to review and manage prescription medications. Nonetheless, our estimates of costs suggest that NMP can be cost-saving for both dietitians and TRs in the short, mid and long term. In addition, although there were minimal differences in effectiveness outcomes, our analysis demonstrated no statistically significant differences across patients managed by prescribers and non-prescribers (where a GP manages medication) for either profession.

The NMP literature has largely focused on evaluating the benefits and effectiveness of prescribing without assessing the costs and resource use [23]. Evaluating the effectiveness outcomes without considering the additional intervention costs (e.g. training costs) or reductions in events (for example, the number of referrals to other prescribers, e.g. GPs in this study) does not facilitate the efficient assessment of innovative interventions such as NMP [55]. Despite the increased uptake of NMP and the increasing number of publications in the UK and around the world, there is still very limited information on the cost-effectiveness of NMP by different groups of non-medical professionals. As most cost-effectiveness evidence relates to pharmacists, it is imperative to assess the impact, safety and economic value of prescribing by non-medical prescribers in other professions to inform policy and practice around NMP where it provides value for money [23].

There is also a need for rigorous economic evaluation studies. Only three model-based economic evaluations assessed the cost-effectiveness of prescribing by pharmacists in Canada [56, 57] and Australia [58]. Other studies used a cost-consequence-based approach (with the majority of the studies conducted in the UK) to measure some of the associated costs and outcomes of care provided by nurses, physiotherapist and podiatrist prescribers without considering the costs of training or other opportunity costs, including staff absence from work to attend the training or providing a cost-effectiveness ratio or assessing sources of uncertainty [12, 51, 59, 60].

Our results show that NMP is resource-intensive due to training costs as well as the longer consultation duration that prescribers spend on medication management and review. This is consistent with previous publications, in which prescribing was perceived as costly in other non-medical prescriber groups such as nurses, physiotherapists, and podiatrists [12, 52, 59]. Similar to our findings, other studies, e.g. Black et al. [2022], Courtenay et al. [2015] and Carey et al. [2020], reported that nurse prescribers had longer consultations but sought less assistance from other colleagues and referred patients less frequently compared to non-prescribers [12, 51, 59]. Also, similar to other studies, D-SPs and TR-IPs were found in higher pay bands than non-prescribers. Regardless of this, our analysis suggests these additional costs can be compensated by fewer referrals to other prescribers (e.g., a GP), and dietitian and therapeutic radiographer prescribers are likely to save costs in the short, mid and long term.

Health-related quality of life was the main health outcome evaluated by many studies in the literature. The three studies that showed pharmacist prescribing is cost-effective used specific effectiveness outcomes (e.g. systolic blood pressure) for patients with specific health conditions (e.g. stroke) [56–58]. Also, the effectiveness outcomes in these studies were observed or modelled over a longer period of time (e.g. 30 years) and compared with the baseline values for the same groups of patients. We were not able to consider specific outcome measures in the model, as there was high heterogeneity in the study participants recruited through multiple practice sites of care. A mixed-effects regression, however, was performed to adjust for the likely random effect of the practice site (different care settings) on the model parameters.

The evidence generated in this study demonstrates there were minimal, statistically non-significant differences in health-related quality of life of patients across prescriber and non-prescriber groups in both professions. Similarly, our findings demonstrate there are no statistically significant differences in ‘patient satisfaction’ or ‘experience of the consultations’ among patients managed by prescribers and non-prescribers (where a GP manages medication) for either profession. No other studies have reported negative impacts on patients’ satisfaction with care, medication, and well-being in other NMP groups [12, 56 – 58, 60]. In the absence of negative outcomes, extending non-medical professionals, such as dietitians’ and TRs’, scope of practice to include supplementary and independent prescribing is key to supporting effective and sustainable delivery of the NHS Long Term Plan [19, 61, 62] and making a step towards capacity and capability development of the workforce to deliver innovative models of service delivery by supporting higher level of clinical autonomy in their advanced practice roles [12, 61].

### Strengths and limitations of the study

This is the only study to date that included the most comprehensive economic assessment of prescribing authority (including costs of training courses, the opportunity cost of time spent on training and longer consultation times by prescribers) in specific groups of non-medical professionals. The decision analytic model developed in the study can be used for planning purposes. The model is user-friendly and allows changing the number of prescribers, the cost of training, the number of consultations, the number of prescriptions, and the number of referrals to other specialists for prescribing. The model can estimate cost savings (losses) and cost-effectiveness of supplementary or independent prescribing in a particular setting in the short and long term.

There are several limitations to this economic evaluation. First, the study experienced a decrease in recruitment and delays in data collection due to coinciding with COVID-19. Second, the original intention was to include the cost of deprescribing^8^ in the model [63]. Deprescribing can potentially reduce the costs of prescribing. However, there was insufficient data to make accurate estimates of the extent or the costs of deprescribing medicines for both professions. Third, a potential advantage of NMP by healthcare professionals is patient access to timely care and medication. Due to a relatively small number of responses, we were unable to produce reliable estimates of the waiting times to obtain a prescription, so this was not included in the model. Fourth, having specific health measures would possibly provide more granular data. Due to high heterogeneity in the study sample (i.e. two professions managing patients with different health conditions), we only considered QALY, a generic health measure, to compare the health outcomes of patients managed by prescribers and non-prescribers. Fifth, the studies that evaluated prescribing rights by non-medical staff used different effectiveness outcomes with varied ranges of costs or comparators (e.g. GPs or non-prescribers) for a range of health conditions, which limits their generalisability, usefulness and comparability with this study and other professions (including dietitians and TRs) and settings [23].

## Conclusion

Despite uncertainties around NMP being cost-effective, the evidence generated in this study suggests that NMP can save costs and resources in the medium to long term. Our findings show that the costs associated with training in the first year and longer consultations by NMP can be compensated by fewer referrals to other prescribers (e.g. GPs and hospital consultants), leading to cost-savings over time. Such long-term savings combined with the healthcare budgetary constraints, chronic shortages of GPs and consultants and the absence of negative impacts on patients’ quality of life and satisfaction is encouraging. Given the ageing population, increasing demand for access to medicine, and a global shortage of health workforce, these findings have important funding implications for national and international policy-makers regarding training and commissioning further prescribing authorities. Future research is needed for a more focused examination of the costs and specific effectiveness outcomes, considering additional sub-groups of patients and practice settings at larger scales to determine the cost-effectiveness of prescribing practices within each profession. This would enable a more robust evidence-based assessment of effectiveness and cost-effectiveness and inform future decisions regarding prescribing by other groups of healthcare professionals.

## Electronic supplementary material

Below is the link to the electronic supplementary material.

Supplementary Material 1

## Data Availability

Data and the model will be made available on request.

## Footnotes

1 The quality, appropriateness and safety of medicines management decisions made during the consultations were assessed as part of the broader project of TRaDiP using case-record reviews and only one medication error (dietitian supplementary prescribing) related to a wrong dose was identified.

2 The Health and Care Professions Council, formerly the Health Professions Council, is a statutory regulator of over 280,000 from 15 health and care professions in the UK.

3 QALY was used as the primary outcome since no participants in a critical condition were included in the study and therefore no mortality was expected. In real life, prescribing for such patients would be managed by a clinical team rather than non-medical prescribers only.

4 QALY values can be also negative when a health state is deemed worse than dead.

5 The personal study time were costed using the average pay band (see Methods for more information).

6 These changes included issuing new prescriptions, changing existing prescriptions, or de-prescribing.

7 The values were adjusted for covariates using a mixed-effects linear model (see Methods for more information).

8 Deprescribing is part of the prescribing process, and it is defined as discontinuing or reducing the dose of medications or changing or stopping medicines.

## References

[1] Marengoni, A., Angleman, S., Melis, R., et al.: Aging with multimorbidity: A systematic review of the literature. Ageing Res. Rev. **10**(4), 430–439 (2011). 10.1016/j.arr.2011.03.003 21402176 10.1016/j.arr.2011.03.003

[2] CDC. Therapeutic Drug Use: Center for Disease Control and Prevention (CDC) National Centre for Health Statistics. (2023). https://www.cdc.gov/nchs/fastats/drug-use-therapeutic.htm Accessed 29 July 2024

[3] NHS Digital: Prescriptions Dispensed in the Community - Statistics for England, 2007–2017. National Health Service Digital. (2018). https://digital.nhs.uk/data-and-information/publications/statistical/prescriptions-dispensed-in-the-community/prescriptions-dispensed-in-the-community-england---2007---2017 Accessed 26 July 2024

[4] NHS Digital: Electronic prescriptions reach one billion a year. National Health Service Digital. (2023). https://digital.nhs.uk/news/2023/electronic-prescriptions-reach-one-billion-a-year Accessed 29 July 2024

[5] Tichy, E.M., Hoffman, J.M., Suda, K.J., et al.: National trends in prescription drug expenditures and projections for 2022. Am. J. Health Syst. Pharm. **79**(14), 1158–1172 (2022). 10.1093/ajhp/zxac102 35385103 10.1093/ajhp/zxac102 PMC9383648

[6] Alves-Conceicao, V., Rocha, K.S.S., Silva, F.V.N., et al.: Are clinical outcomes associated with medication regimen complexity?? A systematic review and Meta-analysis. Ann. Pharmacother. **54**(4), 301–313 (2020). 10.1177/1060028019886846 31718244 10.1177/1060028019886846

[7] Boniol, M., Kunjumen, T., Nair, T.S., et al.: The global health workforce stock and distribution in 2020 and 2030: A threat to equity and ‘universal’ health coverage? BMJ Glob Health. **7**(6) (2022). 10.1136/bmjgh-2022-009316 10.1136/bmjgh-2022-009316 PMC9237893 35760437

[8] Mann, C., Timmons, S., Evans, C., et al.: Exploring the role of advanced clinical practitioners (ACPs) and their contribution to health services in england: A qualitative exploratory study. Nurse Educ. Pract. **67**, 103546 (2023). 10.1016/j.nepr.2023.103546 36739736 10.1016/j.nepr.2023.103546 PMC9872859

[9] Carey, N., Edwards, J., Babashahi, S., Stenner, K.: Medicines management activity of advanced practice dietitians and therapeutic radiographers: A rapid review. J. Prescribing Pract. **12**(6) (2024). 10.12968/jprp.2024.0009

[10] Eddison, N., Healy, A., Darke, N., et al.: Exploration of the representation of the allied health professions in senior leadership positions in the UK National health service. BMJ Lead. **8**(2), 119–126 (2024). 10.1136/leader-2023-000737 37620124 10.1136/leader-2023-000737 PMC12038103

[11] Stewart-Lord, A., Beanlands, C., Khine, R., et al.: The role and development of advanced clinical practice within allied health professions: A mixed method study. J. Multidiscip Health. **13**, 1705–1715 (2020). 10.2147/JMDH.S267083 10.2147/JMDH.S267083 PMC7701658 33268992

[12] Carey, N., Edwards, J., Otter, S., et al.: A comparative case study of prescribing and non-prescribing physiotherapists and podiatrists. BMC Health Serv. Res. **20**(1), 1074 (2020). 10.1186/s12913-020-05918-8 33234141 10.1186/s12913-020-05918-8 PMC7687831

[13] Armstrong, A., Manfrin, A., Gibson, J.: Non-medical prescribing in primary care in the united kingdom: An overview of the current literature. J. Prescribing Pract. **3**(9), 352–361 (2021). 10.12968/jprp.2021.3.9.352

[14] Edwards, J., Coward, M., Carey, N.: Barriers and facilitators to implementation of non-medical independent prescribing in primary care in the UK: A qualitative systematic review. BMJ Open. **12**(6), e052227 (2022). 10.1136/bmjopen-2021-052227 35676011 10.1136/bmjopen-2021-052227 PMC9185484

[15] Armstrong, A.: Non-medical prescribing in primary care in the UK: An overview of the current literature. J. Prescribing Pract. **5**(1) (2023). 10.12968/jprp.2023.5.1.18

[16] Raghunandan, R., Marra, C.A., Tordoff, J., Smith, A.: Examining non-medical prescribing trends in new zealand: 2016–2020. BMC Health Serv. Res. **21**(1), 418 (2021). 10.1186/s12913-021-06435-y 33941188 10.1186/s12913-021-06435-y PMC8094524

[17] Bhanbhro, S., Drennan, V.M., Grant, R., Harris, R.: Assessing the contribution of prescribing in primary care by nurses and professionals allied to medicine: A systematic review of literature. BMC Health Serv. Res. **11**(1), 330 (2011). 10.1186/1472-6963-11-330 22136294 10.1186/1472-6963-11-330 PMC3248914

[18] Department of Health: The National Health System plan: A plan for investment. A plan for reform. Department of Health: London. (2000). http://1nj5ms2lli5hdggbe3mm7ms5.wpengine.netdna-cdn.com/files/2010/03/pnsuk1.pdf Accessed 12 August 2021

[19] National Health Service England. NHS Long Term Workforce Plan: NHS England. (2023). https://www.england.nhs.uk/publication/nhs-long-term-workforce-plan/ Accessed 9 September 2024

[20] Department of Health: Framing the contribution of allied health professions: delivering high quality healthcare. (2008). https://nchalliedhealthprofessionals.wordpress.com/wp-content/uploads/2008/11/framing-the-contribution-of-allied-health-professionals.pdf Accessed 12 November 2024

[21] Cope, L.C., Abuzour, A.S., Tully, M.P.: Nonmedical prescribing: Where are we now? Ther. Adv. Drug Saf. **7**(4), 165–172 (2016). 10.1177/2042098616646726 27493720 10.1177/2042098616646726 PMC4959632

[22] Department of Health and Social Security (DHSS): Medicinal Products; Prescriptions by Nurses Act. (1992). https://www.legislation.gov.uk/ukpga/1992/28/contents Accessed 12 November 2024

[23] Babashahi, S., Carey, N., Jani, Y., et al.: Costs, consequences and value for money in non-medical prescribing: A scoping review. BMJ Open. **13**(5), e067907 (2023). 10.1136/bmjopen-2022-067907 37130673 10.1136/bmjopen-2022-067907 PMC10163523

[24] Cooper, R., Guillaume, L., Avery, T., et al.: Nonmedical prescribing in the united kingdom: Developments and stakeholder interests. J. Ambul. Care Manage. **31**(3), 244–252 (2008). 10.1097/01.JAC.0000324670.91153.b4 10.1097/01.JAC.0000324670.91153.b4 18574383

[25] Graham-Clarke, E., Rushton, A., Noblet, T., Marriott, J.: Non-medical prescribing in the united Kingdom National health service: A systematic policy review. PLoS One. **14**(7), e0214630 (2019). 10.1371/journal.pone.0214630 31356615 10.1371/journal.pone.0214630 PMC6663007

[26] Kroezen, M., van Dijk, L., Groenewegen, P.P., Francke, A.L.: Nurse prescribing of medicines in Western European and Anglo-Saxon countries: A systematic review of the literature. BMC Health Serv. Res. **11**, 127 (2011). 10.1186/1472-6963-11-127 21619565 10.1186/1472-6963-11-127 PMC3141384

[27] Courtenay, M., Carey, N., Stenner, K.: Non medical prescribing leads views on their role and the implementation of Non medical prescribing from a multi-organisational perspective. BMC Health Serv. Res. **11**, 142 (2011). 10.1186/1472-6963-11-142 21635744 10.1186/1472-6963-11-142 PMC3120647

[28] Wilson, D., Murphy, J., Nam, M., et al.: Nurse and midwifery prescribing in ireland: A scope-of-practice development for worldwide consideration. Nurs. Health Sci. **20**, 264–270 (2018). 10.1111/nhs.12408 29377551 10.1111/nhs.12408

[29] Babashahi, S., Carey, N., Jani, Y.H., Hounsome, N.: Costs and consequences of services provided by non-medical prescribers: A scoping review protocol. J. Prescribing Pract. **4**(4), 160–164 (2022). 10.12968/jprp.2022.4.4.160

[30] Health and Care Professions Council: Non-medical prescribing approved courses. Health and Care Professions Council. (2021). https://www.hcpc-uk.org/education/approved-programmes/. Accessed 10 August 2021

[31] NHS England: Workforce, training and education. Training for non-medical prescribers. National Health System England. (2024). https://www.hee.nhs.uk/our-work/medicines-optimisation/training-non-medical-prescribers#:~:text=Is%20funding%20available%20for%20NMP,use%20their%20allocation%20of%20funding Accessed 6 September 2024

[32] General Pharmaceutical Council: Discussion paper on supervising pharmacist independent prescribers in training. London: GPhC. (2016). https://www.the-pda.org/wp-content/uploads/discussion_paper_on_supervising_pharmacist_independent_prescribers_in_training_november_2016_1.pdf Accessed 12 November 2024

[33] Nursing and Midwifery Council: Standards for prescribing programmes (2018). https://www.nmc.org.uk/standards/standards-for-post-registration/standards-for-prescribers/standards-for-prescribing-programmes/ Accessed 12 November 2024

[34] General Optical Council: Independent Prescribing. (2021). https://optical.org/media/j51li2rq/independent-prescribing-handbook.pdf?docid=9B627708-5D4E-48AF-AACCD07F79427B19 Accessed 16 November 2021

[35] Sharman, A.: Independent prescribing: A journey to provide the best possible care. J. Paramedic Pract. **7**(5), 228–232 (2015). 10.12968/jpar.2015.7.5.228

[36] Weeks, G., George, J., Maclure, K., Stewart, D.: Non-medical prescribing versus medical prescribing for acute and chronic disease management in primary and secondary care. Cochrane Database Syst. Rev. **11**(11), CD011227 (2016). 10.1002/14651858.CD011227.pub2 27873322 10.1002/14651858.CD011227.pub2 PMC6464275

[37] Department of Health: Improving patients’ access to medicines: A guide to implementing nurse and pharmacist independent prescribing within the NHS in England. London: Department of Health. (2006). http://webarchive.nationalarchives.gov.uk/ Accessed 15 July 2021

[38] National Health Service England: Allied health professions medicines project. London: National Health System England: (2016). https://www.england.nhs.uk/ahp/med-project/ Accessed 15 August 2021

[39] National Health Service: Non-medical prescribing by allied health professionals. NHS England. (2016). https://www.england.nhs.uk/ahp/med-project/ Accessed 15 August 2021

[40] AHP Medicines Project Team: Summary of the responses to the public consultation on proposals to introduce independent prescribing by radiographers across the United Kingdom. London: NHS England. (2016). https://www.england.nhs.uk/wp-content/uploads/2016/02/radiographers-summary-consult-responses.pdf Accessed 15 August 2021

[41] AHP Medicines Project Team: Summary of the responses to the public consultation on proposals to introduce supplementary prescribing by Dietitians across the United Kingdom. London: NHS England. (2016). https://www.england.nhs.uk/wp-content/uploads/2016/02/dietitians-summary-consult-responses.pdf Accessed 15 August 2021

[42] Noblet, T., Marriott, J., Graham-Clarke, E., et al.: Clinical and cost-effectiveness of non-medical prescribing: A systematic review of randomised controlled trials. PLoS One. **13**(3), e0193286 (2018). 10.1371/journal.pone.0193286 29509763 10.1371/journal.pone.0193286 PMC5839564

[43] TRaDiP research project full report: Evaluation of supplementary prescribing by dietitians and independent prescribing by radiographers. (2024). https://www.surrey.ac.uk/research-projects/evaluation-supplementary-prescribing-dietitians-and-independent-prescribing-radiographers#outputs. Accessed 12

[44] Alghamdi, S.S.A., Hodson, K., Deslandes, P., et al.: Prescribing trends over time by non-medical independent prescribers in primary care settings across Wales (2011–2018): A secondary database analysis. BMJ Open. **10**(10), e036379 (2020). 10.1136/bmjopen-2019-036379 33051229 10.1136/bmjopen-2019-036379 PMC7554451

[45] Herdman, M., Gudex, C., Lloyd, A., et al.: Development and preliminary testing of the new five-level version of EQ-5D (EQ-5D-5L). Qual. Life Res. **20**(10), 1727–1736 (2011). 10.1007/s11136-011-9903-x 21479777 10.1007/s11136-011-9903-x PMC3220807

[46] Jones, K., Weatherly, H., Birch, S., et al.: Unit Costs of Health and Social Care 2022 Manual. Technical report. Personal Social Services Research Unit (University of Kent) & Centre for Health Economics (University of York), Kent, UK. (2023). https://kar.kent.ac.uk/100519/

[47] NHS Digital: The National Cost Collection for the NHS 2021/22. England National Health Service Digital. (2022). https://www.england.nhs.uk/costing-in-the-nhs/national-cost-collection/

[48] NHS: NHS Employers. Annual pay scales 2021/22. National Health Service. (2022). https://www.nhsemployers.org/articles/annual-pay-scales-202122

[49] National Institute for Health and Care Excellence (NICE): NICE health technology evaluations: the manual. National Institute for Health and Care Excellence. (2022). Available from https://www.nice.org.uk/process/pmg36/chapter/introduction-to-health-technology-evaluation

[50] Claxton, K., Paulden, M., Gravelle, H., et al.: Discounting and decision making in the economic evaluation of health-care technologies. Health Econ. **20**(1), 2–15 (2011). 10.1002/hec.1612 21154521 10.1002/hec.1612

[51] Black, A., Courtenay, M., Norton, C., et al.: Independent nurse medication provision: A mixed method study assessing impact on patients’ experience, processes, and costs in sexual health clinics. J. Adv. Nurs. **78**(1), 239–251 (2022). 10.1111/jan.15075 34652029 10.1111/jan.15075

[52] EuroQol Research Foundation: EQ-5D-5L. EuroQol Research Foundation. (2019). Available from https://euroqol.org/information-and-support/euroqol-instruments/eq-5d-5l/

[53] Rabin, R., de Charro, F.: EQ-5D: A measure of health status from the EuroQol group. Ann. Med. **33**(5), 337–343 (2001). 10.3109/07853890109002087 11491192 10.3109/07853890109002087

[54] Devlin, N.J., Shah, K.K., Feng, Y., et al.: Valuing health-related quality of life: An EQ-5D-5L value set for England. Health Econ. **27**(1), 7–22 (2018). 10.1002/hec.3564 28833869 10.1002/hec.3564 PMC6680214

[55] Michelly Goncalves Brandao, S., Brunner-La Rocca, H.P., Pedroso de Lima, A.C., Alcides Bocchi, E.: A review of cost-effectiveness analysis: From theory to clinical practice. Med. (Baltim). **102**(42), e35614 (2023). 10.1097/MD.0000000000035614 10.1097/MD.0000000000035614 PMC10589545 37861539

[56] Al Hamarneh, Y.N., Johnston, K., Marra, C.A., Tsuyuki, R.T.: Pharmacist prescribing and care improves cardiovascular risk, but is it cost-effective? A cost-effectiveness analysis of the RxEACH study. Can. Pharm. J. (Ott). **152**(4), 257–266 (2019). 10.1177/1715163519851822 31320960 10.1177/1715163519851822 PMC6610508

[57] Marra, C., Johnston, K., Santschi, V., Tsuyuki, R.T.: Cost-effectiveness of pharmacist care for managing hypertension in Canada. Can. Pharm. J. (Ott). **150**(3), 184–197 (2017). 10.1177/1715163517701109 28507654 10.1177/1715163517701109 PMC5415065

[58] Hale, A., Merlo, G., Nissen, L., et al.: Cost-effectiveness analysis of doctor-pharmacist collaborative prescribing for venous thromboembolism in high risk surgical patients. BMC Health Serv. Res. **18**(1), 749 (2018). 10.1186/s12913-018-3557-0 30285744 10.1186/s12913-018-3557-0 PMC6167876

[59] Courtenay, M., Carey, N., Gage, H., et al.: A comparison of prescribing and non-prescribing nurses in the management of people with diabetes. J. Adv. Nurs. **71**(12), 2950–2964 (2015). 10.1111/jan.12757 26387971 10.1111/jan.12757

[60] Norman, I.J., Coster, S., McCrone, P., et al.: A comparison of the clinical effectiveness and costs of mental health nurse supplementary prescribing and independent medical prescribing: A post-test control group study. BMC Health Serv. Res. **10**, 4 (2010). 10.1186/1472-6963-10-4 20051131 10.1186/1472-6963-10-4 PMC2820038

[61] Beech, J., Bottery, S., Charlesworth, A., et al.: Closing the gap: Key areas for action on the health and care workforce. The Health Foundation; The Kings Fund & Nuffield Trust. (2019). https://www.kingsfund.org.uk/insight-and-analysis/reports/closing-gap-health-care-workforce

[62] Imison, C., Castle-Clarke, S., Watson, R.: Reshaping the workforce to deliver the care patients need. Research Report. Nuffield Trust. (2016). https://www.nuffieldtrust.org.uk/research/reshaping-the-workforce-to-deliver-the-care-patients-need

[63] Le Bosquet, K., Barnett, N., Minshull, J., Deprescribing: Practical ways to support Person-Centred. Evidence-Based Deprescribing Pharm. (Basel). **7**(3) (2019). 10.3390/pharmacy7030129 10.3390/pharmacy7030129 PMC6789835 31484305

