# Supplementary material for: A model-based cost-effectiveness analysis of prescribing by dietitians and therapeutic radiographers in England
Source: Eur J Health Econ. 2025 Jul 3;27(1):117–33. doi: 10.1007/s10198-025-01813-3 (PMC12929314; doi:10.1007/s10198-025-01813-3)
Supplement: Supplementary file 1 — Supplementary Material 1 [file 10198_2025_1813_MOESM1_ESM.docx]

# **Supplementary Materials**

**Appendix 1: Overview of TRaDiP research project design**

This economic evaluation study is part of a larger research project, ‘the evaluation of supplementary prescribing by dietitians and independent prescribing by radiographers’ (TRaDiP project), funded by the National Institute for Health and Care Research (NIHR) Policy Research Programme. TRaDiP was a mixed-method study undertaken in four phases from 2019–2024 (see <https://www.surrey.ac.uk/research-projects/evaluation-supplementary-prescribing-dietitians-and-independent-prescribing-radiographers#outputs> for more information about TRaDiP). The four phases are briefly described below:

**Phase 1:** Literature review to determine medicines management activity, evidence of effectiveness and barriers and facilitators in practice.

**Phase 2**: Surveys of National Health System (NHS) trusts across England to assess dietitian supplementary prescribing (D-SP) and therapeutic radiographer independent prescribing (TR-IP) to explore prescribing activity and trends over 18 months and identify innovative service models. This phase included surveys with 59 NHS Trust service managers (D=30, TR=29), with follow-up interviews (n=6 per group) 18-22 months later. Also, two surveys were undertaken with prescribers in both professions. A total of 92 prescribers (D=38, TR=54) respondents completed survey 1 and 34 (D=16, TR=18) and survey 2 around 18 months later.

**Phase 3**: This phase included several data collections via self-report audit, interviews, documentary evidence, observations, patient questionnaires, case record review and economic assessment as described briefly below. A comparative case study with economic analysis was conducted across eight sites (eight D-SPs and TR-IPs and eight non-prescribers) in seven geographical locations. Health economic analysis examined the costs, effectiveness, and cost-effectiveness of care delivery for D-SP and TR-IP compared with non-prescription sites (more information is provided in the paper). A total of 513 self-report audits (169 dietitians and 344 TRs) were completed. Interviews were conducted with 15 dietitians and TRs, as well as 18 team members across the study case sites. Patient questionnaires were completed by a total of 180 patients managed by 49 dietitians and 131 TRs. A total of 32 case record reviews were assessed for ten dietitians and 22 TRs.

**Phase 4:** Development of an online prescribing toolkit. A toolkit, co-produced with patients, was developed to support healthcare workers in getting the most out of the prescribing qualification.

**Appendix 2: List of model parameters used in the study**

| **Parameter** | **Value** | | **Reference** |
| --- | --- | --- | --- |
|  | **Prescriber** | **Non-prescriber** |  |
| **Dietitians** | | | |
| ***Probability of using their prescribing rights*** | | | |
| base case | 0.64 | N/A | Data from the study sample |
| lower limit | 0.34 | N/A | Assumption (±30% from the base case) |
| upper limit | 0.94 | N/A | Assumption (±30% from the base case) |
| ***Probability of referring patients for prescribing to other prescribers*** | | | |
| base case | 0.02 | 0.30 | Data from the study sample |
| lower limit | 0.00 | 0.05 | Data from the study sample |
| upper limit | 0.32 | 0.60 | Assumption (+30% from the base case) for the prescribers and data from the study sample for the non-prescribers |
| ***Probability of prescribing not being required*** | | | |
| base case | 0.34 | 0.70 | Data from the study sample |
| lower limit | 0.04 | 0.40 | Assumption (±30% from the base case) |
| upper limit | 0.64 | 1.00 | Assumption (±30% from the base case) |
| ***QALY*** | | | |
| base case | 0.74 | 0.75 | Data from the study sample |
| lower limit | 0.70 | 0.71 | Data from the study sample |
| upper limit | 0.78 | 0.82 | Data from the study sample |
| **Therapeutic radiographers** | | | |
| ***Probability of using their prescribing rights*** | | | |
| base case | 0.87 | N/A | Data from the study sample |
| lower limit | 0.57 | N/A | Assumption (±30% from the base case) |
| upper limit | 1.00 | N/A | Assumption (±30% from the base case) |
| ***Probability of referring patients to other prescribers*** | | | |
| base case | 0.07 | 0.23 | Data from the study sample |
| lower limit | 0.01 | 0.09 | Data from the study sample |
| upper limit | 0.10 | 0.37 | Data from the study sample |
| ***Probability of prescribing not being required*** | | | |
| base case | 0.06 | 0.77 | Data from the study sample |
| lower limit | 0.06 | 0.47 | Assumption (±30% from the base case) |
| upper limit | 0.36 | 1.00 | Assumption (±30% from the base case) |
| ***QALY*** | | | |
| base case | 0.73 | 0.74 | Data from the study sample |
| lower limit | 0.68 | 0.69 | Data from the study sample |
| upper limit | 0.78 | 0.82 | Data from the study sample |

Note: N/A: Not Applicable; QALY: Quality-adjusted life year.

**Appendix 3.1: List of training courses included in health economics analysis**

| **No** | **Name of course** | **Dietitians, TRs, or both** | **Provider** | **Course duration (months)** | **Source** |
| --- | --- | --- | --- | --- | --- |
| 1 | Independent/Supplementary Prescribing (PG Cert) | Both | University of Kent | 10 | https://www.kent.ac.uk/courses/postgraduate/740/independent-supplementary-prescribing |
| 2 | Independent and Supplementary Prescribing | Both | Liverpool John Moores University | 6.5 | https://www.ljmu.ac.uk/study/cpd/stand-alone-cpd-courses/non-medical-prescribing |
| 3 | Continuing Professional Development (CPD)/Short courses (Practice Certificate in Independent and Supplementary Prescribing – L6) | Both | Coventry University | 3.25 | <https://www.coventry.ac.uk/course-structure/health-and-life-sciences/cpd/practice-certificate-in-independent-and-supplementary-prescribing-level-6/> |
| 4 | Non-Medical Prescribing (Independent and/or Supplementary Prescribing) | Both | University of the West of England, Bristol, in partnership with the University of Bath | 7 | https://courses.uwe.ac.uk/Z51000077/non-medical-prescribing-independent-andor-supplementary-prescribing |
| 5 | Non-medical prescribing CPD award | Both | University of the Highlands and Islands | 5 | https://www.uhi.ac.uk/en/courses/cpd-award-non-medical-prescribing/#tabanchor |
| 6 | Independent/Supplementary Prescribing (V300) L7 | Both | De Montfort University, Leicester | 7 | https://www.dmu.ac.uk/study/courses/postgraduate-courses/independent-supplementary-prescribing-v300/independent-supplementary-prescribing-v300-level-7.aspx |
| 7 | Postgraduate certificate in non-medical prescribing (PG Cert NMP) | Both | The Open University | 13 | http://www.open.ac.uk/postgraduate/qualifications/k33 |
| 8 | Non-Medical Prescribing | Both | Anglia Ruskin University | 6 | https://aru.ac.uk/study/professional-and-short-courses/v300-non-medical-prescribing |
| 9 | Non-Medical Prescribing (V300) - Level 7 | Both | Sheffield Hallam University | 2.5 | https://www.shu.ac.uk/study-here/options/health-and-social-care/short-courses-and-modules/nonmedical-prescribing-v300--level-7 |
| 10 | Non-Medical Prescribing (V300) - Level 6 | Both | Northumbria University | 5.5 | https://www.northumbria.ac.uk/study-at-northumbria/continuing-professional-development-short-courses-specialist-training/non-medical-prescribing-v300---level-6---ac0636-ac0637/ |
| 11 | Advanced Certificate Non-Medical Prescribing | Both | University of Central Lancashire | 6 | https://www.uclan.ac.uk/cpd/courses/non-medical-prescribing-advcert |
| 12 | Advancing Non-medical Prescribing | Both | Teesside University | 4.5 | https://www.tees.ac.uk/parttime_courses/nursing_&_health/ucppd_advancing_non-medical_prescribing.cfm |
| 13 | Non-Medical Prescribing Programme | Both | University of Northampton | 6 | https://www.northampton.ac.uk/courses/nonmedical-prescribing-programmes/ |
| 14 | Non-medical Prescribing (V300) | Both | Manchester Metropolitan University | 6 | https://www.mmu.ac.uk/hpsc/cpd/course/non-medical-prescribing-v300/ |
| 15 | Non-Medical Prescribing (V300) | Both | Edge Hill University | 6.5 | https://www.edgehill.ac.uk/health/cpd-modules/non-medical-prescribing-v300-2/ |
| 16 | Non-Medical Prescribing | Both | University of Chester | 6 | https://www1.chester.ac.uk/study/postgraduate/non-medical-prescribing-chester |
| 17 | Independent/Supplementary Prescribing (V300) | Both | University of Surrey | 6 | https://www.surrey.ac.uk/cpd-and-short-courses/independentsupplementary-prescribing-v300-level-7 |
| 18 | Independent Prescribing | Both | University of Brighton | 6 | https://www.brighton.ac.uk/studying-here/find-a-course/cpe-in-health-sciences/undergraduate/modules/independent-prescribing-for-nurses-midwives-allied-health-professionals.aspx |
| 19 | Non-medical prescribing | Both | Bucks New University | 7.5 | https://bucks.ac.uk/courses/short-course/non-medical-prescribing-formerly-independent-and-supplementary-nurse-prescribing |
| 20 | Independent and Supplementary Prescribing for Healthcare Professionals | Both | Edinburgh Napier University | 6.5 | https://www.napier.ac.uk/about-us/our-schools/the-school-of-health-and-social-care/courses/independent-and-supplementary-prescribing |

Note: TRs: Therapeutic radiographers. Data on courses were collected in January–March 2021.

**Appendix 3.2: Summary of the characteristics of 20 NMP training programmes* in the UK in 2021**

| **Item** | **Mean (range)** |
| --- | --- |
| Duration (week) | 6 (3–13) |
| Number of training sessions (day) | 30 (10–48) |
| Class/study time (hour) | 7.5 (7–8) |
| Fee (£) | 1,800 (1,220–3,240) |
| Course credits | 40 (20–60) |
| Annual intake | 2 (2–4) |
| Average number of attendees per intake | 60 (40–80) |

*All data were collected from the websites of sample courses approved by the Health and Care Professions Council for dietitians and therapeutic radiographers [18]. A few course organisers were contacted to obtain information on the annual intake and the average number of attendees per intake.

**Appendix 4: List of assumptions used in the economic analysis**

| **Item** | **Assumption** |
| --- | --- |
| Cost of the patient referral to other prescribers (including doctors) | Patients’ referrals to other prescribers were costed in line with the NICE recommendations as these usually mean unplanned consultations, which cannot be used for other patients. We assumed that 50% of referrals would be managed face-to-face and 50% via telephone or online consultation. |
| The training course fee | An average course fee was estimated based on data from the study sample and data gathered from the Health and Care Professions Council website and course programme webpages (see Supplementary Information, Tables S3.1 and S3.2) for both professions. |
| OOP expenses | The OOP expenses related to the training programme (i.e. travel, accommodation, study material, etc) paid by the professionals were calculated based on data from the study sample. Two cost scenarios were assumed: one including the OOP expenses (as the employers might pay a refund for this) and one excluding these expenses. |
| Cost of time off work to complete the course | The costs associated with time off work were derived from data collected for the study sample and estimated using the NHS pay scales [35]. |
| Cost of personal study time | The data collected during the study shows that the trained professionals were reimbursed partly for their personal study time during the training course, which was included in the non-medical prescribing training-related costs. Where the professionals were not reimbursed for their personal study time, it was considered as part of the OOP expenses incurred by the professionals. |
| Patient contact | The average number of patient contacts per year was estimated using the data collected for the study sample. We assumed a year of 48 working weeks for both professions to calculate the number of patient contacts annually (<https://www.healthcareers.nhs.uk/explore-roles/allied-health-professionals/roles-allied-health-professions/>). |
| Safety and medical errors | We assumed safety is consistent between both arms of the model. In real life, prescribing for critically ill patients would be managed by a clinical team rather than non-medical prescribers alone. In addition, the quality, appropriateness and safety of medicines management decisions made during the consultations were assessed as part of the broader project of TRaDiP using case-record reviews, and only one medication error (dietitian supplementary prescribing) related to a wrong dose was identified. |
| Compliance | The model incorporated compliance using the probability of ‘prescribing rights used’. Compliance was measured using data from the study questionnaires (for more information, see the Model structure in the Methods section, Appendix 14, and Appendix 15). |

Note: NICE: National Institute for Health and Care Excellence; OOP: Out-of-pocket expenses

**Appendix 5: Unit costs used in the analysis**

| **Service code** | **Service description** | **Currency code** | **Currency description** | **National average unit cost** |
| --- | --- | --- | --- | --- |
| 100 | General Surgery Service - Consultant Led | WF01A | Non-Admitted Face-to-Face Attendance, Follow-up | £162.93 |
| 100 | General Surgery Service - Consultant Led | WF01B | Non-Admitted Face-to-Face Attendance, First | £216.38 |
| 100 | General Surgery Service - Consultant Led | WF01C | Non-Admitted Non-Face-to-Face Attendance, Follow-up | £135.43 |
| 100 | General Surgery Service - Consultant Led | WF01D | Non-Admitted Non-Face-to-Face Attendance, First | £149.17 |
| 654 | Dietetics Service - Consultant Led | WF01A | Non-Admitted Face-to-Face Attendance, Follow-up | £98.43 |
| 654 | Dietetics Service - Consultant Led | WF01B | Non-Admitted Face-to-Face Attendance, First | £134.20 |
| 654 | Dietetics Service - Consultant Led | WF01C | Non-Admitted Non-Face-to-Face Attendance, Follow-up | £109.61 |
| 654 | Dietetics Service - Consultant Led | WF01D | Non-Admitted Non-Face-to-Face Attendance, First | £122.24 |
| 811 | Interventional Radiology Service - Consultant Led | WF01A | Non-Admitted Face-to-Face Attendance, First | £126.57 |
| 811 | Interventional Radiology Service - Consultant Led | WF01B | Non-Admitted Non-Face-to-Face Attendance, Follow-up | £93.87 |
| 811 | Interventional Radiology Service - Consultant Led | WF01C | Non-Admitted Non-Face-to-Face Attendance, Follow-up | £52.10 |
| 811 | Interventional Radiology Service - Consultant Led | WF01D | Non-Admitted Non-Face-to-Face Attendance, First | £76.82 |
| 300 | General Internal Medicine Service - Consultant Led | WF01A | Non-Admitted Face-to-Face Attendance, Follow-up | £174.64 |
| 300 | General Internal Medicine Service - Consultant Led | WF01B | Non-Admitted Face-to-Face Attendance, First | £183.84 |
| 300 | General Internal Medicine Service - Consultant Led | WF01C | Non-Admitted Non-Face-to-Face Attendance, Follow-up | £148.70 |
| 300 | General Internal Medicine Service - Consultant Led | WF01D | Non-Admitted Non-Face-to-Face Attendance, First | £146.03 |
| 305 | Clinical Pharmacology Service - Consultant Led | WF01A | Non-Admitted Face-to-Face Attendance, Follow-up | £191.93 |
| 305 | Clinical Pharmacology Service - Consultant Led | WF01B | Non-Admitted Face-to-Face Attendance, First | £326.88 |
| 305 | Clinical Pharmacology Service - Consultant Led | WF01C | Non-Admitted Non-Face-to-Face Attendance, Follow-up | £132.47 |
| 305 | Clinical Pharmacology Service - Consultant Led | WF01D | Non-Admitted Non-Face-to-Face Attendance, First | £168.53 |
| 320 | Cardiology Service - Consultant Led | WF01A | Non-Admitted Face-to-Face Attendance, Follow-up | £186.38 |
| 320 | Cardiology Service - Consultant Led | WF01B | Non-Admitted Face-to-Face Attendance, First | £200.40 |
| 320 | Cardiology Service - Consultant Led | WF01C | Non-Admitted Non-Face-to-Face Attendance, Follow-up | £157.60 |
| 320 | Cardiology Service - Consultant Led | WF01D | Non-Admitted Non-Face-to-Face Attendance, First | £181.04 |
| 340 | Respiratory Medicine Service - Consultant Led | WF01A | Non-Admitted Face-to-Face Attendance, Follow-up | £194.30 |
| 340 | Respiratory Medicine Service - Consultant Led | WF01B | Non-Admitted Face-to-Face Attendance, First | £262.25 |
| 340 | Respiratory Medicine Service - Consultant Led | WF01C | Non-Admitted Non-Face-to-Face Attendance, Follow-up | £148.10 |
| 340 | Respiratory Medicine Service - Consultant Led | WF01D | Non-Admitted Non-Face-to-Face Attendance, First | £174.52 |
| 370 | MEDICAL ONCOLOGY SERVICE - Consultant Led | WF01A | Non-Admitted Face-to-Face Attendance, Follow-up | £221.48 |
| 370 | MEDICAL ONCOLOGY SERVICE - Consultant Led | WF01B | Non-Admitted Face-to-Face Attendance, First | £363.83 |
| 370 | MEDICAL ONCOLOGY SERVICE - Consultant Led | WF01C | Non-Admitted Non-Face-to-Face Attendance, Follow-up | £190.91 |
| 370 | MEDICAL ONCOLOGY SERVICE - Consultant Led | WF01D | Non-Admitted Non-Face-to-Face Attendance, First | £199.69 |
| 720 | Eating Disorders Service - Consultant Led | WF01A | Non-Admitted Face-to-Face Attendance, Follow-up | £183.40 |
| 720 | Eating Disorders Service - Consultant Led | WF01B | Non-Admitted Face-to-Face Attendance, First | £295.15 |
| 720 | Eating Disorders Service - Consultant Led | WF01C | Non-Admitted Non-Face-to-Face Attendance, Follow-up | £183.12 |
| 720 | Eating Disorders Service - Consultant Led | WF01D | Non-Admitted Non-Face-to-Face Attendance, First | £183.40 |
| 800 | Clinical Oncology Service - Consultant Led | WF01A | Non-Admitted Face-to-Face Attendance, Follow-up | £184.97 |
| 800 | Clinical Oncology Service - Consultant Led | WF01B | Non-Admitted Face-to-Face Attendance, First | £302.95 |
| 800 | Clinical Oncology Service - Consultant Led | WF01C | Non-Admitted Non-Face-to-Face Attendance, Follow-up | £84.49 |
| 800 | Clinical Oncology Service - Consultant Led | WF01D | Non-Admitted Non-Face-to-Face Attendance, First | £75.96 |
| 307 | Diabetes Service - Consultant Led | WF01A | Non-Admitted Face-to-Face Attendance, Follow-up | £213.79 |
| 307 | Diabetes Service - Consultant Led | WF01B | Non-Admitted Face-to-Face Attendance, First | £290.27 |
| 307 | Diabetes Service - Consultant Led | WF01C | Non-Admitted Non-Face-to-Face Attendance, Follow-up | £165.04 |
| 307 | Diabetes Service - Consultant Led | WF01D | Non-Admitted Non-Face-to-Face Attendance, First | £189.69 |
| 361 | Renal Medicine Service - Consultant Led | WF01A | Non-Admitted Face-to-Face Attendance, Follow-up | £217.41 |
| 361 | Renal Medicine Service - Consultant Led | WF01B | Non-Admitted Face-to-Face Attendance, First | £284.31 |
| 361 | Renal Medicine Service - Consultant Led | WF01C | Non-Admitted Non-Face-to-Face Attendance, Follow-up | £157.33 |
| 361 | Renal Medicine Service - Consultant Led | WF01D | Non-Admitted Non-Face-to-Face Attendance, First | £192.40 |

Note: The services and their unit costs were extracted from The National Health Reference Cost Collection 2021/22 (accessible from <https://www.england.nhs.uk/costing-in-the-nhs/national-cost-collection/>).

**Appendix 6: Patient contacts and referrals, mean (range)**

|  | **Dietitians** | | **Therapeutic radiographers** | |
| --- | --- | --- | --- | --- |
|  | **Prescribers** | **Non-prescribers** | **Prescribers** | **Non-prescribers** |
| All patient contacts per week | 9 (5–15) | 9 (5–15) | 19 (6–38) | 19 (6–38) |
| % of patient contacts required to manage prescriptions | 63% (50%–75%) | N/A | 63% (41%–90%) | N/A |
| Number of patient contacts required to manage prescriptions per week | 6 (3–9) | N/A | 12 (4–24) | N/A |
| % of referrals for prescribing | 2% (0%–2%) | 30% (5%–60%) | 7% (1%–10%) | 23% (9%–37%) |
| Total number of patient contacts per year | 442 (240–720) | 442 (240–720) | 909 (272–1800) | 909 (272–1800) |
| Number of patient contacts required to manage a prescription per year | 276 (150–450) | N/A | 575 (172–1139) | N/A |
| Number of referrals for prescribing per year | 30 (5–77) | 131 (72–209) | 60 (18–119) | 213 (64–421) |

N/A: Not Applicable; * the figures are rounded up to the nearest whole number, as they show the number of patient contacts.

**Appendix 7: Summary of effectiveness outcomes (non-adjusted)**

| **Assessment** | **Prescribers** | | **Non-prescribers** | | **Difference in mean** | **95% CI** |
| --- | --- | --- | --- | --- | --- | --- |
|  | **Mean** | **SD** | **Mean** | **SD** |  |  |
| **Dietitians** | | | | | | |
| QALY | 0.6267 | 0.3068 | 0.6414 | 0.2961 | 0.0147 | -0.1587–0.1881 |
| Patient’s overall satisfaction with consultation* | 77 | 7 | 76 | 8 | 1 | -3–5 |
| Patient’s overall experience of the consultation* | 65 | 7 | 63 | 3 | 2 | -2–6 |
| **Therapeutic radiographers** | | | | | | |
| QALY | 0.8337 | 0.1297 | 0.8430 | 0.1574 | 0.0092 | -0.0416–0.0601 |
| Patient’s overall satisfaction with consultation* | 79 | 8 | 80 | 7 | 1 | -2–2 |
| Patient’s overall experience of the consultation* | 66 | 7 | 66 | 5 | 0 | -2–2 |

Note: * Patients’ satisfaction and experience outcomes scores were estimated using a 100 scale.

**Appendix 8: EQ-5D-5L responses for patients managed by dietitians and therapeutic radiographers**

| **Dimensions** | **Dietitians** | | **Therapeutic radiographers** | |
| --- | --- | --- | --- | --- |
|  | **Prescriber group (n=25)**  **N (%)** | **Non-prescriber group (n=24)**  **N (%)** | **Prescriber group (n=57)**  **N (%)** | **Non-prescriber group (n=74)**  **N (%)** |
| **Mobility** | | |  |  |
| No problems | 5 (20%) | 7 (29%) | 48 (84%) | 65 (88%) |
| Slight | 6 (24%) | 6 (25%) | 7 (12%) | 7 (10%) |
| Moderate | 7 (28%) | 4 (17%) | 1 (2%) | 1 (1%) |
| Severe | 4 (16%) | 4 (17%) | 1 (2%) | 1 (1%) |
| Extreme | 3 (12%) | 3 (13%) | 0 (0%) | 0 (0%) |
| **Self-care** | | |  |  |
| No problems | 16 (64%) | 15 (63%) | 51 (89%) | 66 (89%) |
| Slight | 2 (8%) | 3 (13%) | 4 (7%) | 6 (8%) |
| Moderate | 6 (24%) | 4 (17%) | 1 (2%) | 2 (3%) |
| Severe | 0 (0%) | 1 (4%) | 1 (2%) | 0 (0%) |
| Extreme | 1 (4%) | 1 (4%) | 0 (0%) | 0 (0%) |
| **Usual activities** | | |  |  |
| No problems | 5 (20% | 5 (21%) | 22 (38.5%) | 44 (60%) |
| Slight | 6 (24%) | 6 (25%) | 21 (37%) | 11 (15%) |
| Moderate | 8 (32%) | 8 (33%) | 11 (19%) | 17 (23%) |
| Severe | 4 (16%) | 3 (13%) | 1 (2%) | 1 (1%) |
| Extreme | 2 (8%) | 2 (8%) | 2 (3.5%) | 1 (1%) |
| **Pain/Discomfort** | | |  |  |
| No pain | 7 (28%) | 7 (29%) | 16 (28%) | 26 (35%) |
| Slight | 7 (28%) | 6 (25%) | 23 (40%) | 32 (43%) |
| Moderate | 7 (28%) | 5 (21%) | 14 (25%) | 9 (12%) |
| Severe | 3 (12%) | 5 (21%) | 4 (7%) | 5 (7%) |
| Extreme | 1 (4%) | 1 (4%) | 0 (0%) | 2 (3%) |
| **Anxiety/Depression** | | |  |  |
| No problems | 12 (48%) | 15 (63%) | 32 (56%) | 46 (62%) |
| Slight | 7 (28%) | 3 (13%) | 17 (30%) | 22 (30%) |
| Moderate | 2 (8%) | 3 (13%) | 7 (12%) | 6 (8%) |
| Severe | 1 (4%) | 2 (8%) | 1 (2%) | 0 (0%) |
| Extreme | 3 (12%) | 1 (4%) | 0 (0%) | 0 (0%) |

**Appendix 9: Patient waiting time to obtain a prescription (day) for the two professions**

|  | **Prescribers** | | **Non-prescribers** | |
| --- | --- | --- | --- | --- |
|  | **N*** | **Mean (SD)** | **N** | **Mean (SD)** |
| Waiting time for patients managed by dietitians | 5 | 1.67 (0.6) | 3 | 3.7 (2.3) |
| Waiting time for patients managed by therapeutic radiographers | 12 | 1 (N/A) | 3 | 1 (N/A) |

Note: * Sample size for this effectiveness outcome; N/A: Not Applicable.

**Appendix 10: Cost-effectiveness plane for dietitian prescribers vs dietitian non-prescribers based on 5,000 Monte Carlo simulations of total costs and patient overall satisfaction with the consultation**

**Appendix 11: Cost-effectiveness plane for dietitian prescribers vs dietitian non-prescribers based on 5,000 Monte Carlo simulations of total costs and patient overall experience of the consultation**

**Appendix 12: Cost-effectiveness plane for therapeutic radiographer prescriber vs non-prescribers based on 5,000 Monte Carlo simulations of total costs and patient overall satisfaction with the consultation**

**Appendix 13: Cost-effectiveness plane for therapeutic radiographer prescriber vs non-prescribers based on 5,000 Monte Carlo simulations of total costs and patient overall experience of the consultation**

**Appendix 14: Deterministic sensitivity analyses of supplementary prescribing by dietitians**

|  | | **Difference in cost, £ (95%CI)** | **Difference in QALY**  **(95%CI)** | **ICER point estimate, £**  **(95%CI)** |
| --- | --- | --- | --- | --- |
| **Base case** | | -10 (-179–120) | -0.0122 (-0.0824–0.0566) | 816 (-25,289–23,788) |
| **Parameter** | | | | |
| **Prescriber** | **Non-prescriber** |  | | |
| **QALY** | | | | |
| base case: 0.74 | base case: 0.75 | -10 (-179–120) | -0.0122 (-0.0824–0.0566) | 816 (-25,289–23,788) |
| lower limit: 0.70 | lower limit: 0.71 | -10 (-179–120) | -0.0100 (-0.0787–0.0577) | 993 (-25,486–25,450) |
| upper limit: 0.78 | upper limit: 0.82 | -10 (-179–120) | -0.0400 (-0.1080–0.0290) | 248 (-17,446–14,445) |
| **Probability of using prescribing rights** | | | | |
| base case: 0.64 | N/A | -10 (-179–120) | -0.0122 (-0.0824–0.0566) | 816 (-25,289–23,788) |
| lower limit: 0.34 | N/A | -20 (-184–112) | -0.0122 (-0.0824–0.0566) | 1,643 (-20,988–24,892) |
| upper limit: 0.94 | N/A | 1 (-166–138) | -0.0122 (-0.0824–0.0566) | -82 (-29,177–26,208) |
| **Probability of prescribing not being required** | | | | |
| base case: 0.34 | base case: 0.70 | -10 (-179–120) | -0.0122 (-0.0824–0.0566) | 816 (-25,289–23,788) |
| lower limit: 0.04 | lower limit: 0.40 | -72 (-261–73) | -0.0122 (-0.0824–0.0566) | 11.996 (-40,066–37,090) |
| upper limit: 0.64 | upper limit: 1.00 | -34 (-268–137) | -0.0122 (-0.0824–0.0566) | 5,665 (-39,420–37,056) |
| **Probability of referring patients to other prescribers** | | | | |
| base case: 0.02 | base case: 0.30 | -10 (-179–120) | -0.0122 (-0.0824–0.0566) | 816 (-25,289–23,788) |
| lower limit: 0.00 | lower limit: 0.05 | 34 (-57–120) | -0.0122 (-0.0824–0.0566) | -2,793 (-21,115–19,512) |
| upper limit: 0.32 | upper limit: 0.60 | -10 (-309–248) | -0.0122 (-0.0824–0.0566) | 821 (-50,616–43,994) |
| **Cost of non-medical prescribing, excluding OOP expenses per patient contact** | | | | |
| base case: £12 | N/A | -19 (-190–114) | -0.0122 (-0.0824–0.0566) | 1,590 (-27,372, 25,794) |
| lower limit: £10 | N/A | -21 (-195–110) | -0.0122 (-0.0824–0.0566) | 1,755 (-24,821–29,635) |
| upper limit: £16 | N/A | -15 (-182–119) | -0.0122 (-0.0824–0.0566) | 1,262 (-26,333–29,607) |
| **Cost of non-medical prescribing, including OOP expenses per patient contact** | | | | |
| base case: £21 | N/A | -10 (-179–120) | -0.0122 (-0.0824–0.0566) | 816 (-25,289–23,788) |
| lower limit: £20 | N/A | -11 (-183–115) | -0.0122 (-0.0824–0.0566) | 933 (-28,251–24,793) |
| upper limit: £23 | N/A | -8 (-176–124) | -0.0122 (-0.0824–0.0566) | 687 (-28,889–24,668) |
| **Cost of referral for prescribing per patient contact** | | | | |
| base case: £188 | base case: £188 | -10 (-179–120) | -0.0122 (-0.0824–0.0566) | 816 (-25,289–23,788) |
| lower limit: £76 | lower limit: £76 | 14 (-223–161) | -0.0122 (-0.0824–0.0566) | 1,150 (-33,798–35,502) |
| upper limit: £364 | upper limit: £364 | -95 (-300–70) | -0.0122 (-0.0824–0.0566) | 7,803 (3,591–6,421) |
| **Cost of consultation (and prescribing-related activities) per patient contact** | | | | |
| base case: £157 | base case: £123 | -10 (-179–120) | -0.0122 (-0.0824–0.0566) | 816 (-25,289–23,788) |
| lower limit: £125 | lower limit: £98 | -24 (-231–127) | -0.0122 (-0.0824–0.0566) | 1,971 (-28,894–31,417) |
| upper limit: £190 | upper limit: £149 | -33 (-227–119) | -0.0122 (-0.0824–0.0566) | 2,711 (-32,360–31,977) |

Note: *Cost per QALY lost. QALY, Quality-adjusted life years; ICER, Incremental cost-effectiveness ratio; prescribing; NMP, non-medical prescribing; OOP, Out-of-pocket.

**Appendix 15: Deterministic sensitivity analyses of independent prescribing by therapeutic radiographers**

|  | | **Difference in cost, £ (95%CI)** | **Difference in QALY**  **(95%CI)** | **ICER point estimate, £**  **(95%CI)** |
| --- | --- | --- | --- | --- |
| **Base case** | | 5 (-194–183) | -0.0060 (-0.0816–0.0686) | -824 (-37,645–24,032) |
| **Parameter** | | | | |
| **Prescriber** | **Non-prescriber** |  | | |
| **QALY** | | | | |
| base case: 0.73 | base case: 0.74 | 5 (-194–183) | -0.0060 (-0.0816, 0.0686) | -824 (-37,645–24,032) |
| lower limit: 0.68 | lower limit: 0.69 | 5 (-194–183) | -0.0100 (-0.0866, 0.0661) | -494 (-28,950–23,313) |
| upper limit: 0.78 | upper limit: 0.82 | 5 (-194–183) | -0.0400 (-0.1178, 0.0371) | -124 (-18,064–17,804) |
| **Probability of using prescribing rights** | | | | |
| base case: 0.87 | N/A | 5 (-194–183) | -0.0060 (-0.0816, 0.0686) | -824 (-37,645–24,032) |
| lower limit: 0.57 | N/A | -4 (-204–167) | -0.0060 (-0.0816, 0.0686) | 596 (-32,474–24,538) |
| upper limit: 1.00 | N/A | -3 (-192–163) | -0.0060 (-0.0816, 0.0686) | 525 (-30,775–25,650) |
| **Probability of prescribing not being required** | | | | |
| base case: 0.06 | base case: 0.77 | 5 (-194–183) | -0.0060 (-0.0816, 0.0686) | -824 (-37,645–24,032) |
| lower limit: 0.06 | lower limit: 0.47 | 32 (-145–206) | -0.0060 (-0.0816, 0.0686) | -5,347 (-26,514–27,906) |
| upper limit: 0.36 | upper limit: 1.00 | 15 (-213–231) | -0.0060 (-0.0816, 0.0686) | -2,499 (-34,980–35,429) |
| **Probability of referring patients to other prescribers** | | | | |
| base case: 0.07 | base case: 0.23 | 5 (-194–183) | -0.0060 (-0.0816, 0.0686) | -824 (-37,645–24,032) |
| lower limit: 0.01 | lower limit: 0.09 | 21 (-127–168) | -0.0060 (-0.0816, 0.0686) | -3,499 (-20,499–21,765) |
| upper limit: 0.10 | upper limit: 0.37 | -13 (-127–168) | -0.0060 (-0.0816, 0.0686) | 2,166 (-38,176–34,147) |
| **Cost of non-medical prescribing, excluding OOP expenses per patient contact** | | | | |
| base case: £6 | N/A | 2 (-194–187) | -0.0060 (-0.0816, 0.0686) | -308 (-30,366–32,210) |
| lower limit: £5 | N/A | 1 (-198–184) | -0.0060 (-0.0816, 0.0686) | -142(-30,986–30,498) |
| upper limit: £14 | N/A | 10 (-189–197) | -0.0060 (-0.0816, 0.0686) | -1,641 (-27,2313–34,675) |
| **Cost of non-medical prescribing, including OOP expenses per patient contact** | | | | |
| base case: £10 | N/A | 5 (-194–183) | -0.0060 (-0.0816, 0.0686) | -824 (-37,645–24,032) |
| lower limit: £10 | N/A | 5 (-194–183) | -0.0060 (-0.0816, 0.0686) | -824 (-37,645–24,032) |
| upper limit: £16 | N/A | 12 (183–193) | -0.0060 (-0.0816, 0.0686) | -1,975 (-29,524–34,404) |
| **Cost of referral for prescribing per patient contact** | | | | |
| base case: £179 | base case: £179 | 5 (-194–183) | -0.0060 (-0.0816, 0.0686) | -824 (-37,645–24,032) |
| lower limit: £76 | lower limit: £76 | 23 (-190–183) | -0.0060 (-0.0816, 0.0686) | -3,832 (-33,467–29,009) |
| upper limit: £364 | upper limit: £364 | -23 (-226–155) | -0.0060 (-0.0816, 0.0686) | 3,832 (-24,980–29,418) |
| **Cost of consultation (and prescribing-related activities) per patient contact** | | | | |
| base case: £116 | base case: £87 | 5 (-194–183) | -0.0060 (-0.0816, 0.0686) | -824 (-37,645–24,032) |
| lower limit: £69 | lower limit: £52 | 1 (-218–204) | -0.0060 (-0.0816, 0.0686) | -5,347 (-26,514–27,906) |
| upper limit: £168 | upper limit: £127 | 15 (-213–231) | -0.0060 (-0.0816, 0.0686) | -2,499 (-34,980–35,429) |

*Cost per QALY lost. QALY, Quality-adjusted life years; ICER, Incremental cost-effectiveness ratio; prescribing; NMP, non-medical prescribing; OOP, Out-of-pocket.
